# Exploring tumor clonal evolution in bone marrow of patients with diffuse large B-cell lymphoma by deep *IGH* sequencing and its potential relevance in relapse

**DOI:** 10.1038/s41408-019-0229-1

**Published:** 2019-08-21

**Authors:** Jiao Ma, David Redmond, Ayako Miyaguchi, Anna S. Nam, Kui Nie, Susan Mathew, Olivier Elemento, Wayne Tam

**Affiliations:** 1 Department of Biochemistry & Molecular Cell Biology, Shanghai Jiaotong University, School of Medicine, Shanghai, China; 2 Department of Pathology and Laboratory Medicine, Weill Cornell Medicine, New York, NY USA; 3 Department of Physiology and Biophysics & Institute for Computational Biomedicine, Weill Cornell Medicine, New York, NY USA

**Keywords:** B-cell lymphoma, B-cell lymphoma

Dear Editor,

Diffuse large B-cell lymphoma (DLBCL) is the most common lymphoma and accounts for 30–40% of all B-cell non-Hodgkin lymphoma (NHLs)^1,2^. Many patients respond to the first-line regimens such as R-CHOP^3^. However, up to one-third of patients have refractory and relapse disease associated with poor outcome. DLBCL is heterogeneous with respect to morphologic, immunophenotypic, and molecular features^4^, and encompasses numerous variants, subgroups, and subtypes. Approximately 10–15% of DLBCL patients have bone marrow (BM) involvement^5–7^. Morphologically, lymphoma involvement in the BM of DLBCL patients can either be classified as large B-cell lymphoma (histologically concordant) or indolent, low-grade B-cell lymphoma (histologically discordant).

Tumor clonal dynamics and evolution in the BM of patients with DLBCL have not yet been studied in detail. Questions remain whether the lymphomatous involvement in BM in DLBCL patients represents a direct dissemination of tumor cells from the dominant tumor clones in the nodal or extranodal location to the BM. It is also unknown whether tumor-related cells can be detected in the BM when there is no morphologic evidence of lymphoma, and whether detection of these cells can have prognostic utility. Deep immunoglobulin heavy-chain gene enables the study of tumor evolution in B-cell malignancies, and has been previously employed in elucidating the patterns of clonal evolution in DLBCL relapse^8^ based on a combination of VDJ determination, somatic hypermutation profiling, and phylogenetic analysis.

In our study, we selected 28 DLBCL cases with paired staging bone marrows (BM) (Supplementary Table 1). *IGH* VDJ deep sequencing and phylogenetic analysis were performed (Supplementary Figs. 1, 2) on the diagnostic DLBCL samples and their matched BM samples (*n* = 29). In addition, two patients (#22 and #45) had posttreatment BM only, and are included for separate case analysis (see below). The dominant rearrangements identified in the DLBCL are listed in Supplementary Table 2. The phylogenetic relationships between the dominant tumor clones of the diagnostic DLBCLs and the tumor-related clones identified in the corresponding staging BM samples are summarized in the heatmap (Fig. 1a, b), and representative phylogenetic trees are shown (Fig. 1c).

**Fig. 1:**
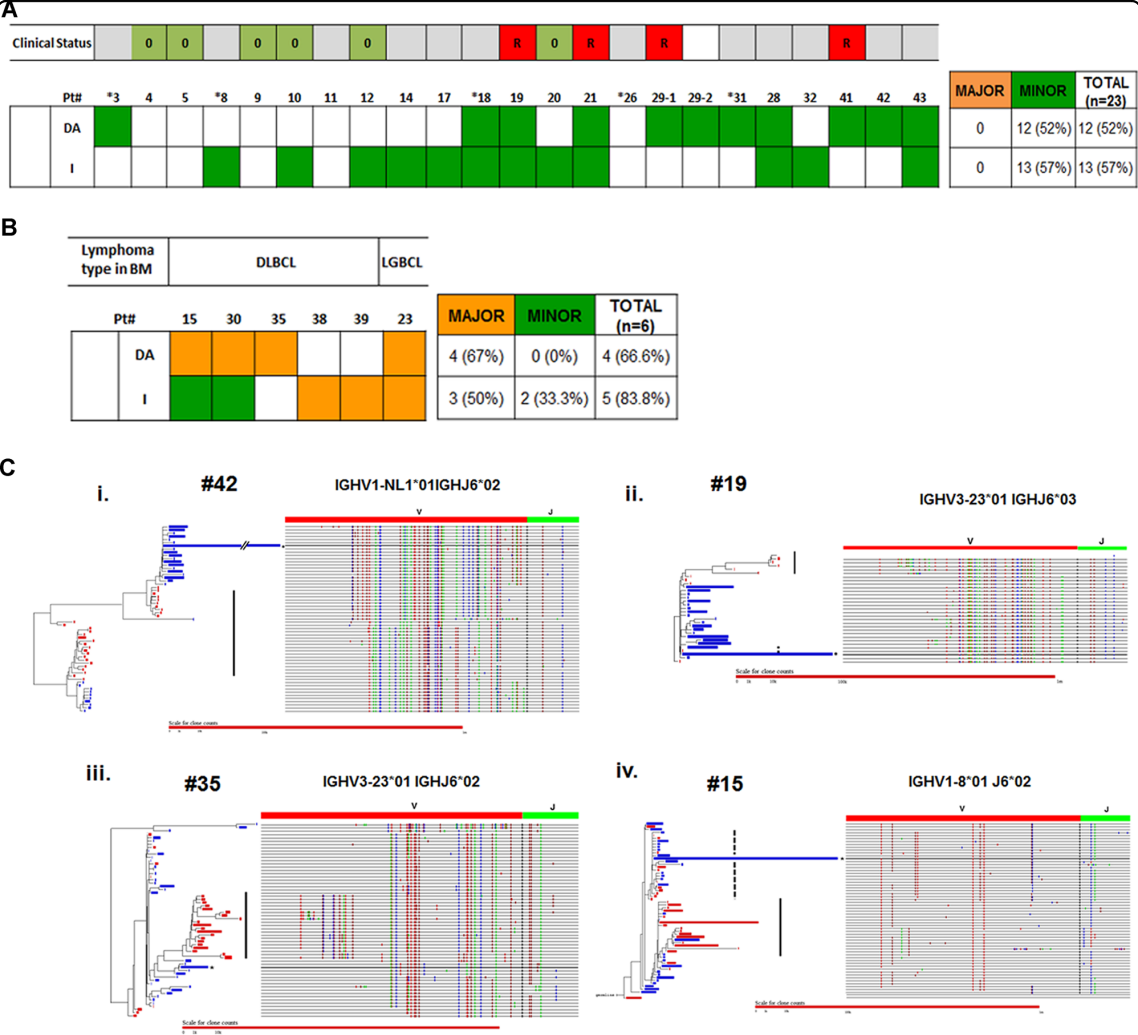
Summary of types of tumor-related clones in the staging bone marrow of patients with DLBCL. The patients are categorized into (**a**) those with morphologic evidence of lymphoma (DLBCL, diffuse large B-cell lymphoma or LGBCL, low-grade B-cell lymphoma) in the BM; (**b**) those with no detectable morphologic evidence of lymphoma. For each DLBCL-BM pair, the tumor-related clones in the BM are further sub-classified as divergent/ancestral (hereby designated as “DA”) if the SHM patterns of the tumor clones in the marrow demonstrate divergence or appear ancestral relative to the dominant lymphoma clone in the diagnostic DLBCL. They are sub-classified as identical (designated as “I”) if they showed a SHM pattern that was identical or immediately descendant to the dominant lymphoma clone in the diagnostic DLBCL. In addition, a major clone is defined as a clone for which the VDJ rearrangement is the most abundant among other VDJ rearrangements; whereas a minor clone is defined as a clone not representing the most abundant VDJ rearrangement. The presence and subtype(s) of tumor-related clones (DA = divergent or ancestral, I = identical) in the BM for each patient is indicated in a heatmap-like chart. Orange denotes that the clones in the BM are major, and green denotes that the tumor clones in the marrow are minor. Cases without any detectable tumor clones are shown as blank. In five of these 23 samples without morphologic evidence of lymphoma (#28, 32, 41, 42, and 43), flow cytometric abnormalities were detected. VDJ analysis identified dominant B-cell clones unrelated to the DLBCL tumor clones in four of the five cases (see Supplementary Table 2B), implying that the abnormalities seen in the flow cytometry do not represent tumor involvement in the BM but instead are incidental findings. In the remaining case (#41), no dominant B-cell clone was detected in the BM; however, CLL-like B-cells were detected by flow cytometry, which are most consistent with monoclonal B-cell lymphocytosis of undetermined significance (CLL-type). Patients in which comparisons were made between relapsed DLBCL samples and matched staging BM samples because of the unavailability of the original diagnostic DLBCL were marked with asterisks. For patients with no morphologic evidence of lymphoma involvement in BM, the presence or absence (indicated by R or 0 in the clinical status) of subsequent relapse was indicated. Patients who was lost to follow-up or did not have staging BM samples at diagnosis are excluded (colored gray). **c** Representative phylogenetic trees comparing tumor-related clones detected in the bone marrow and in the diffuse large B-cell lymphomas present in the lymph nodes. The dominant tumor clones identified in the DLBCL are marked with asterisks. BM tumor clones identical to the dominant DLBCL clones in the LN (I-type clones, also see Fig. 1) are marked with dashed lines. BM tumor clones divergent or ancestral to the dominant DLBCL clones in the LN (DA-type clones) are marked with solid lines. Sequence reads derived from the LN and BM are indicated in blue and red, respectively, and the lengths of the horizontal bars reflect the numbers of reads. The somatic hypermutation profile in the *IHG* VJ region (right) corresponding to each horizontal bar (left) is shown, with each nucleotide mismatch from the germline sequences indicated by a vertical colored bar. Representative figures in four different cases are shown (i–iv). (i) No histologic evidence of lymphoma in the BM (Pt. #42). Minor DA tumor clones are detected. (ii) No histologic evidence of lymphoma in the BM (Pt. #19). Both minor DA and I clones are detected. (iii) BM involvement by DLBCL (pt. #35). Tumor clones of the DA-type are identified. (iv) BM involvement by DLBCL (Pt. #15). The DA tumor clones predominate in the BM, and I-type clones are present in relatively low abundance

Minor tumor-related clones were detected in 19 (82.6%) of the 23 BM samples which did not exhibit morphologic evidence of lymphomatous involvement. These analyzes demonstrate that these minor clones are present in the BM in minute quantity, ranging from ~0.01% to 1.98%. (Supplementary Table 2A). Among the minor tumor clones identified in these 23 BM samples, 6 (26.1%) were divergent/ancestral (DA)-type only, 7 (30.4%) were identical (I)-type only and 5 (21.7%) were both DA and I. No tumor clones were detected in five BM samples. Our results indicate frequent minimal disease involvement by tumor-related clones detectable by deep IGH sequencing in morphologically normal BM of patients with DLBCL.

We also studied the clonal patterns in BM with morphologic evidence of involvement. Major clones were detected in all six BM specimens with morphologic evidence of lymphoma (DLBCL or low-grade B-cell lymphoma) (Fig. 1b; Supplementary Table 2C). One of them (16.7%) is of DA-type only, and two are of I-type only (33.3%). Minor I-type clones were also found in two of the three BM cases with major DA-type clones. The BM sample with low-grade B-cell lymphoma harbored I-type and DA-type tumor clones of approximately equal abundance. No significant difference in distribution of the DA and I clone types was observed between the BM with or without morphologic evidence of lymphoma.

As DLBCL relapses occur primarily via divergent evolution^8^, we hypothesize that there may be an association between the presence of DA tumor clones in the BM and subsequent relapse. Among the 22 DLBCL patients with no morphologic evidence of involvement, 12 had clinical follow-up. Four of these 12 patients relapsed with DLBCL. Interestingly, DA-type minor tumor clones were detected, either solely or with I-type clones, in the staging marrow of all four patients. For the other eight patients who did not relapse, DA-type clones were detected in only two of them. None of the six patients without detectable tumor clones or I-type clone only in the BM developed relapse (Supplementary Table 3). These findings suggest a possible association of detection of minor DA-type tumor clones in the staging marrow with subsequent relapse (*p* = 0.06, Fisher’s exact test).

In our cohort, we identified three patients (#29, #45, and #22) who had posttransplant DLBCL relapse (Supplementary Table 1). Patient #29 serves as an excellent example to illustrate a direct link between these minor DA clones in the BM and subsequent DLBCL relapse (Fig. 2a). Both the pre-transplant and posttransplant BMs showed no morphologic evidence of lymphoma or flow cytometry abnormalities. However, we identified in the staging BM a minor DA-type clone which persisted in the posttransplant BM, implying chemoresistance. Importantly, this DA clone has an identical SHM pattern to the dominant tumor clone in the relapsed DLBCL, and therefore represents the actual relapse precursor clone (pre-R).

**Fig. 2:**
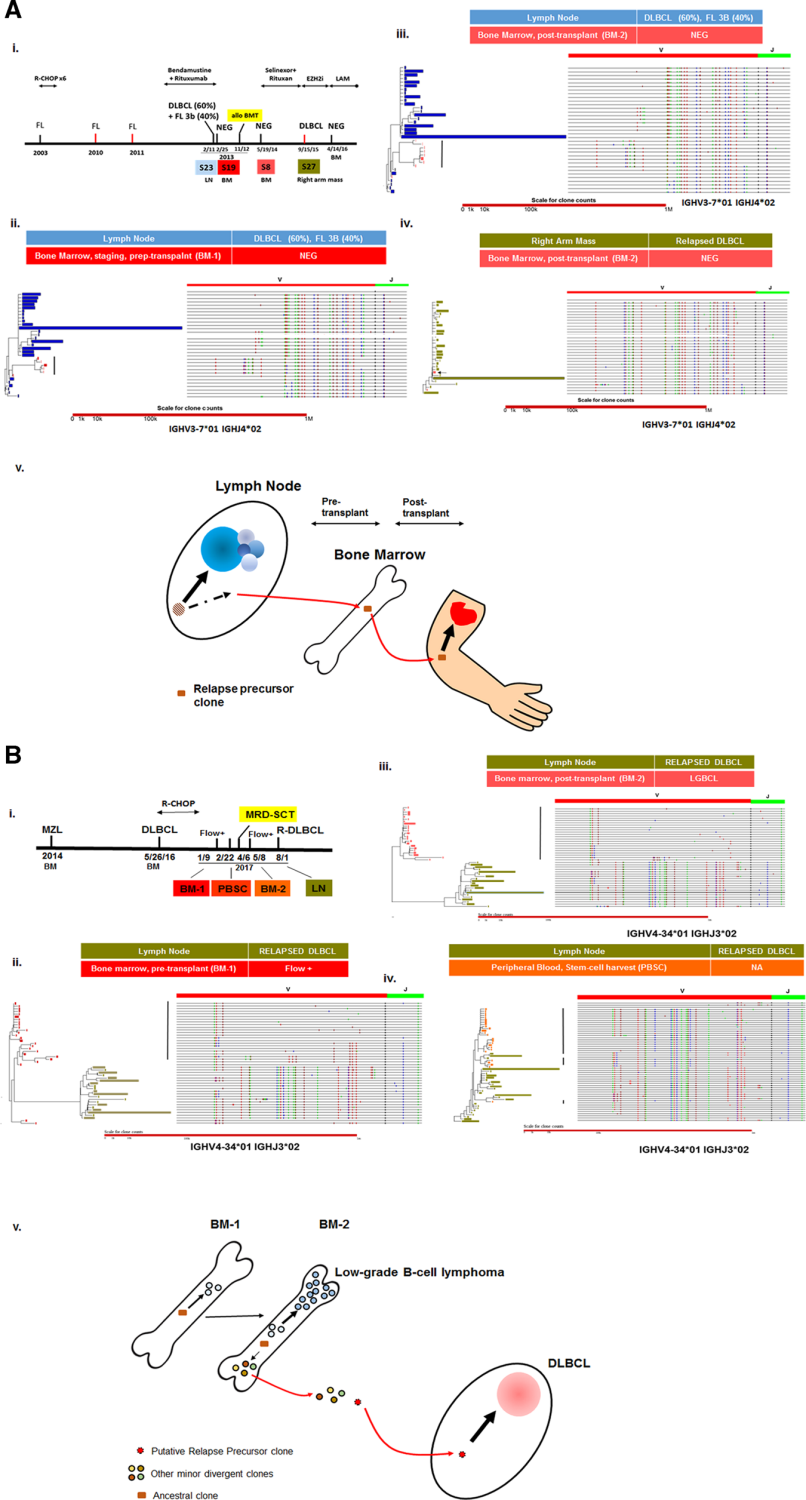
Relapse precursor clones may arise from minor DA-type tumor clones detected in BM. **a** Identification of a minute relapse precursor clone in the post-transplant BM. (i) Timeline of diagnoses, samples and treatment. The patient (#29) had a history of follicular lymphoma (FL), previously treated, and presented with DLBCL and FL, grade 3B. The staging BM was negative. The patient then had an allogenic BM transplant. A BM sample taken 9 months after BM transplant was negative. The patient relapsed with DLBCL as an arm mass. (ii) Comparison of the deep *IGH* sequencing data between the primary LN and the negative pre-transplant staging BM revealed minor divergent clones (highlighted by solid vertical bar). (iii) The minor divergent clones detected in (ii) appeared to persist in the posttransplant BM, suggesting that they are chemoresistant. (iv) A minor divergent clone detected in (ii) and (iii) was identical to the major tumor clone in the relapsed DLBCL (marked by arrow), suggesting that it serves as a relapse precursor clone. (v) A hypothetical model illustrating how a minor divergent clone derived from a putative ancestral clone may serve as relapse precursor. **b** Identification of chemoresistant tumor clones ancestral or highly related to the relapse clones in posttreatment BM and peripheral blood. In patient #45, only the relapsed DLBCL in the LN was available for analysis; the pre-treatment DLBCL was unavailable. BM-1, post chemotherapy, pre-transplant BM; BM-2, posttransplant BM. PBSC, peripheral blood stem cell harvest. There was no morphologic evidence of lymphoma in BM-1, but flow cytometry detected a small monotypic B-cell population. BM-2 showed a low-grade B-cell lymphoma (LGBCL) morphologically. The timeline of the diagnoses, samples and treatment for Patient #45 is indicated in (i). (ii)-(iv) demonstrates the phylogenetic comparisons between BM-1, BM-2, PBSC, and the relapsed DLBCL by deep IGH VJ sequencing. Ancestral tumor clones were identified in BM-1 and BM-2 (ii & iii), and based on their IGH somatic hypermutation patterns, they have the potential to give rise to the relapse precursor clone. Minor divergent tumor clones closely related to the dominant relapsed DLBCL clones were identified in PBSC (iv), but the exact relapse precursor clone could not be identified. Since all these tumor clones in the BM were detected in posttreatment samples, they are likely to be chemoresistant. (v). A hypothetical model linking the detected DA clones and relapse in DLBCL in case #45. Ancestral, presumably chemoresistant clones persist in both the post-chemotherapy, pre-transplant BM (BM-1) as well as the posttransplant BM (BM-2). These clones gradually expanded to form frank low-grade B-cell lymphoma (LGBCL) in the posttransplant BM. The ancestral clones also had the capacity to generate the putative relapse precursor clone (pre-R) that eventually gave rise to the relapse DLBCL. However, it was not detected, possibly because of its extremely low abundance. Minor DA clones closely related to the pre-R clones and derived from the ancestral clones could be detected in the peripheral blood

For #45, a pre-transplant marrow taken during remission post R-CHOP was not diagnostic for lymphoma based on morphologic grounds, but flow cytometry identified a small monotypic B-cell population with similar immunophenotypic profile as the diagnostic DLBCL which represented ~2.5% of analyzed cells. Deep VDJ sequencing identified minor DA clones representing ancestral precursors which are potentially capable of generating the pre-R clone. (Fig. 2b). Similar DA clones were also seen in the posttransplant BM involved by a low-grade B-cell lymphoma with ~10–20% of involvement, suggesting that these ancestral minor DA clones are most likely chemoresistant and have expanded to form frank lymphoma in the BM. Interestingly, in the peripheral blood stem cell harvest (45-SC) sample, minor DA clones closely related and slightly divergent to the relapsed DLBCL were detected. However, the actual pre-R clones might be exceedingly rare in the BM or PB and escape detection. Similar clones representing ancestral precursors were also observed in the posttransplant marrow in patient #22 (Supplementary Fig. 3). Patient #22 eventually relapsed; however, the relapse sample was not available for molecular analysis.

Our findings demonstrate the feasibility of this NGS method to detect minimal lymphoma involvement in the marrow. Our analysis based on DLBCL and the corresponding staging marrows revealed that tumor-related clones can be detected frequently in BM without morphologic evidence of lymphoma using this highly sensitive technique. A major finding from our study is the frequent detection of DA-type tumor clones. These findings underscore tumor heterogeneity and also suggest distinct biological properties of these DA tumor clones, which may have higher intrinsic propensity to disseminate to the BM compared with the dominant tumor clones despite their low abundance in the diagnostic tumor. In addition, our study shows that morphologically concordant involvement of the BM does not necessarily imply genetic concordance. Three of the 5 BM involved by DLBCL that were analyzed harbored dominant/major DA-type clones, suggesting that overt BM involvement in patients with DLBCL is not simply a direct extension of the nodal/extranodal DLBCL to the BM but can represent a tumor divergently evolving in parallel to the main tumor. These findings may have important therapeutic implications, as it is conceivable that the tumor in the bone marrow may demonstrate different drug responsiveness from the extramedullary tumors because of the diverse genetic composition.

Significant difference was not detected in the distribution of clonal types (i.e., DA- or I-type) between the involved or uninvolved BM samples based on analysis of a small cohort. Similar patterns of clones can be seen in both involved and uninvolved BM. These observations suggest a model of tumor progression in the BM, initiating from minute tumor-related clones not detected by morphology and expanding to overt lymphoma (Supplementary Fig. 4).

There seems to be a possible association between the detection of minor DA-type clones in the staging BM of patients without evidence of lymphoma involvement, and subsequent relapse. This association is in line with the previous observation that DLBCL relapse occurs predominantly via divergent evolution^8,9^. A statistical trend (*p* ~ 0.06) could be detected based on analysis of a limited number of samples. However, a larger sample size is needed to confirm this association.

Our study of two illustrative cases suggest that identification of minor DA-type clones in the BM/circulation in a posttreatment setting may signify the presence of pre-R (relapse precursor) clones and herald increased risk of relapse. Recent studies have demonstrated the utility of monitoring clonal VDJ rearrangements and gene mutations in cell-free DNA in predicting DLBCL relapse^10–12^. Our assay provides a simple, feasible, and highly sensitive methodology to detect relapse precursor clones directly. Our study serves as a pilot proof-of-principle investigation that suggests serial monitoring of divergent circulating tumor clones from post-therapy bone marrow, peripheral blood or plasma at different time points to decipher clonal dynamics of these clones may be of clinical value to predict relapse. It is conceivable that emergence or rise in the abundance of one of these divergent subclones may serve as a predictive marker of impending relapse. Additional investigation on a larger cohort is necessary to examine in detail the association between the minor post-therapy tumor-related clones, chemoresistance, and subsequent DLBCL relapse.

## Supplementary information

Supplementary table 1

Supplementary table 2

Supplementary table 3

Supplementary figures

Supplementary file and Figure legends
