# Supplementary material for: Exploring tumor clonal evolution in bone marrow of patients with diffuse large B-cell lymphoma by deep IGH sequencing and its potential relevance in relapse
Source: Blood Cancer J. 2019 Aug 21;9(9):69. doi: 10.1038/s41408-019-0229-1 (PMC6704167; doi:10.1038/s41408-019-0229-1)
Supplement: Supplementary file 1 — Supplementary table 1 [file 41408_2019_229_MOESM1_ESM.docx]

| **Patient**  **#** | **Specimen Type** | **Specimen Date** | | **Age/Sex** | | **Morphologic Evidence of Lymphoma Involvement** | | | **Disease** | **Clinical Notes** | | |
| --- | --- | --- | --- | --- | --- | --- | --- | --- | --- | --- | --- | --- |
| **3** | Spleen | 10/3/2012 | | 71/M | |  | | | DLBCL (relapse), non-GCB | History of DLBCL, treated with R-CHOP+ Velcade. CT (6/18/12) shows new splenic lesion, FDG avid. R-DICE, went into CR & auto-transplant on 12/13/12 | | |
|  | BM | 10/17/2012 | |  | | No | | |  | Staging marrow for relapsed lymphoma | | |
| **4** | Lung | 8/20/2010 | | 67/F | |  | | | DLBCL, GCB |  | | |
|  | BM | 8/31/2010 | |  | | No | | |  | Staging marrow | | |
| **5** | LN | 10/9/2009 | | 45/M | |  | | | DLBCL, non-GCB |  | | |
|  | BM | 10/9/2009 | |  | | No | | |  | Staging marrow | | |
| **8** | LN | 10/3/2011 | | 81/M | |  | | | DLBCL, GCB (relapse) | History of DLBCL (12/9/2009) treated with R-CHOP, found to have recurrent disease by PET scan in early 2011 | | |
|  | BM | 4/21/2011 | |  | | No | | |  | Staging marrow | | |
| **9** | Submandibular Mass | | 7/25/2011 | | 76/M | |  | DLBCL, non-GCB | | |  | |
|  | BM | | 4/26/2011 | |  | | No |  | | | Staging marrow | |
| **10** | LN | 9/6/2012 | | 76/M | |  | | | DLBCL, non GCB |  | | |
|  | BM | 9/10/2012 | |  | | No | | |  | Staging marrow | | |
| **11** | LN | 1/5/2012 | | 59/F | |  | | | DLBCL, non-GCB |  | | |
|  | BM | 1/13/2012 | |  | | No | | |  | Staging marrow | | |
| **12** | Inguinal LN | 9/22/2011 | | 47/M | |  | | | DLBCL, GCB |  | | |
|  | BM | 10/3/2011 | |  | | No | | |  | Staging marrow | | |
| **14** | Axillary Mass | 8/18/2010 | | 69/M | |  | | | DLBCL, non-GCB | | |  |
|  | BM | 8/19/2010 | |  | | No | | |  | | | Staging marrow |
| **15** | Axillary LN | 3/26/2010 | | 81/M | |  | | | EBV^+^ DLBCL, non- GCB | | |  |
|  | BM | 3/25/2010 | |  | | DLBCL | | |  | | | Staging marrow |
| **17** | Retroperitoneal LN | 2/22/2010 | | 52/F | |  | | | DLBCL, non GCB | | |  |
|  | BM | 2/18/2010 | |  | | No | | |  | | | Staging marrow |
| **18** | Flank mass | 2/22/2010 | | 68/M | |  | | | DLBCL, GCB (relapse) | | | History of DLBCL in 1998, treated with COD-BLAM. Dx with FL gra1, treated with chemo/XRT, with Rituxan maintenance. 2002 FL and this specimen are not clonally related |
|  | BM | 3/2/2010 | |  | | No | | |  | | | Staging marrow |
| **19** | Mesenteric LN | 8/10/2009 | | 39/M | |  | | | DLBCL monomorphic B cell PTLD, EBV+  non GCB | | | **DLBCL RELAPSE** (6/21/2011) |
|  | BM | 8/26/2009 | |  | | No | | |  | | | Staging marrow |
| **20** | Groin Mass | 3/11/2009 | | 72/F | |  | | | DLBCL, GCB | | |  |
|  | BM | 3/26/2009 | |  | | No | | |  | | | Staging marrow |
| **21** | Neck Mass | 4/4/2008 | | 86/F | |  | | | DLBCL, GCB (75%)  FL 3B (25%) | | | S/p R-CHOP**, DLBCL RELAPSE** (12/9/2011), |
|  | BM | 4/15/2008 | |  | | No | | |  | | | Staging marrow |
| **22** | Spleen | 2/2/2006 | | 62/F | |  | | | DLBCL, GCB | | | Hx of FL and HL  Autologous transplant 8/1/06  **DLBCL RELAPSE** in lung in 1/2007 |
|  | BM | 11/14/2006 | |  | | No | | |  | | | Post-transplant marrow |
| **23** | Spleen | 10/7/2004 | | 64/M | |  | | | DLBCL + CD5+ low-grade B-cell lymphoma, probably CLL/SLL |  | | |
|  | BM | 10/5/2004 | |  | | CD5+ low grade B-cell lymphoma | | |  | Staging marrow | | |
| **26** | LN | 4/29/2015 | | 69/M | |  | | | DLBCL w/ plasmablastic differentiation, non-GCB (relapse) | s/p R-CHOP for DLBCL in cervical LN in 2012 | | |
|  | BM | 5/15/2015 | |  | | No | | |  | Staging marrow | | |
| **28** | Stomach, cardiac mass biopsy | 8/6/2015 | | 81/M | |  | | | DLBCL, GCB | Dx with FL in 2011 | | |
|  | BM | 8/7/2015 | |  | | No  Flow+ (Igλ-restricted CD5- CD10- B cells, 5%) | | |  | Staging marrow | | |
| **29** | LN | 2/11/2013 | | 52/M | |  | | | DLBCL (60%) & FL Gr3B (40%) | History of FL, s/p chemo (R-CHOP) in 2003, and Bendamustine/Rituxan in 2012-2013. BM allogenic transplant on 11/12/2013. | | |
|  | BM1 | 2/25/2013 | |  | | No | | |  | Pre-transplant staging marrow | | |
|  | BM2 | 5/19/2014 | |  | | No | | |  | Post-transplant marrow | | |
|  | Right arm mass | 9/15/2015 | |  | |  | | | DLBCL, GCB (relapse) | **DLBCL RELAPSE** | | |
| **30** | LN | 9/24/2015 | | 59/F | |  | | | DLBCL (20%) & FL Gr3B (80%) |  | | |
|  | BM | 9/23/2015 | |  | | DLBCL | | |  | Staging marrow | | |
| **31** | LN | 1/22/2014 | | 65/M | |  | | | DLBCL, non-GCB ( relapse) | Hx of Hodgkin in 1970s  DLBCL dx in 2009 treated with R_CHOP, in remission | | |
|  | BM1 | 2/24/2014 | |  | |  | | |  | Staging marrow | | |
| **32** | Testis | 3/11/2016 | | 87/M | |  | | | DLBCL, non-GCB |  | | |
|  | BM | 3/4/2016 | |  | | No  Flow+ (B-cells with decreased κ:λ , 1.6% | | |  |  | | |
| **35** | LN | 12/20/2013 | | 78/M | |  | | | DLBCL (10%), GCB  FL Gr3B (50%),  FL Gr1-2 (40%) |  | | |
|  | BM | 12/19/2013 | |  | | DLBCL | | |  | Staging marrow | | |
| **38** | BM | 9/28/2016 | | 54/M | |  | | | Plasmablastic lymphoma |  | | |
|  | LN | 9/30/2016 | |  | | Plasmablastic lymphoma | | |  | Staging marrow | | |
| **39** | Stomach | 4/13/2016 | | 61/F | |  | | | DLBCL, non-GCB |  | | |
|  | BM | 6/3/2016 | |  | | DLBCL | | |  | Staging marrow | | |
| **41** | LN | 6/1/2016 | | 55/M | |  | | | DLBCL, CD5+ | s/p R-CHOP | | |
|  | BM1 | 5/26/2016 | | 54/M | | No  Flow+  (CLL-like B-cells, 0.2%) | | |  | Staging marrow | | |
|  | Brain | 4/12/2017 | |  | |  | | | DLBCL (relapse), CD5-, non-GCB | **DLBCL RELAPSE** | | |
| **42** | LN | 6/22/2016 | | 73/F | | No  Flow+  (CLL-like B-cells, 0.7%) | | | DLBCL, GCB |  | | |
|  | BM | 6/21/2016 | |  | |  | | |  | Staging marrow | | |
| **43** | LN | 6/30/2016 | | 77/M | |  | | | DLBCL, non-GCB |  | | |
|  | BM | 7/8/2016 | |  | | No  Flow+ (B-cells with decreased κ:λ , 0.8% | | |  | Staging marrow | | |
| **45** | LN | 8/1/2017 | | 69/M | |  | | | DLBCL (relapse), non-GCB | HX of MZL in 2014, dx in BM. DLBCL in BM (5/20/2016). Patient with lymphadenopathy and  Splenomegaly. S/p R-CHOP and went into remission MRD-SCT 4/6/2017 | | |
|  | BM1 | 1/9/2017 | |  | | No  Flow+ (slg-restricted, CD5-CD10+ B cells, 2.7%) | | |  | Pre-transplant marrow | | |
|  | BM2 | 5/8/2017 | |  | | Low-grade B-cell lymphoma | | |  | Post-transplant marrow | | |
|  | SC | 2/22/2017 | |  | | No flow performed | | |  | PB- stem-cell harvest | | |

**Supplementary Table 1. Clinicopathologic characteristics of DLBCL and paired bone marrow samples.** GCB, germinal center B-cell; LN, lymph node; BM, bone marrow.
