# Supplementary material for: Exploring tumor clonal evolution in bone marrow of patients with diffuse large B-cell lymphoma by deep IGH sequencing and its potential relevance in relapse
Source: Blood Cancer J. 2019 Aug 21;9(9):69. doi: 10.1038/s41408-019-0229-1 (PMC6704167; doi:10.1038/s41408-019-0229-1)
Supplement: Supplementary file 2 — Supplementary table 2 [file 41408_2019_229_MOESM2_ESM.docx]

Supplementary Table 2 (A-C)

**Major rearrangements detected in the DLBCL and their abundance in the matched staging marrow with positive DA and/or I clones (see Figure 1).**

| **Patient ID** | **Site** | **Diagnosis** | **Hans COO** | **MAJOR VJ** | **% of total reads**  **of MAJOR VJ**  **in DLBCL or BM** |
| --- | --- | --- | --- | --- | --- |
| **3** | Spleen | DLBCL | Non-GCB | IGHV4-34*01IGHJ2*01 | 97.6 |
|  | BM |  |  | NA | 0.05 |
| **4** | Lung | DLBCL | GCB | IGHV3-d*01 IGHJ4*02 | 23.7 |
|  | BM |  |  | NA | 0.02 |
| **5** | LN | DLBCL | Non-GCB | IGHV6-1*01IGHJ4*02 | 77.8 |
|  | BM |  |  | NA | 0.09 |
| **8** | LN | DLBCL | GCB | IGHV4-39*01IGHJ6*03 | 84.3 |
|  | BM |  |  | NA | 0.09 |
| **10** | LN | DLBCL | Non-GCB | IGHV3-30*02IGHJ4*02 | 93.3 |
|  | BM |  |  | NA | 0.17 |
| **11** | LN | DLBCL | Non-GCB | IGHV3-30*03IGHJ4*02 | 29.6 |
|  | BM |  |  | NA | 1.98 |
| **12** | Inguinal LN | DLBCL | GCB | IGHV4/OR15-8*01IGHJ6*02 | 79.6 |
|  | BM |  |  | NA | 0.02 |
| **14** | Axillary LN | DLBCL | Non-GCB | IGHV4-34*01IGHJ4*02 | 84.7 |
|  | BM |  |  | NA | 0.28 |
| **17** | Retroperitoneal LN | DLBCL | Non-GCB | IGHV3-23*01IGHJ5*01 | 63.6 |
|  | BM |  |  | NA | 0.04 |
| **18** | Flank mass | DLBCL | GCB | IGHV1-3*01IGHJ5*02 | 96.7 |
|  | BM |  |  | NA | 0.18 |
| **19** | Mesenteric LN | DLBCL | Non-GCB | IGHV3-23*01IGHJ6*03 | 92.4 |
|  | BM |  |  | NA | 0.15 |
| **20** | Groin mass | DLBCL | GCB | IGHV4-4*01IGHJ3*02 | 19.1 |
|  | BM |  |  | NA | 0.01 |
| **21** | Neck mass | DLBCL | GCB | IGHV3-7*01IGHJ6*02 | 81.8 |
|  | BM |  |  | NA | 0.66 |
| **22** | Spleen | DLBCL | GCB | IGHV4-34*01IGHJ6*03 | 44.7 |
|  | BM |  |  | NA | 0.04 |
| **26** | LN | DLBCL | Non-GCB | IGHV3-23*01IGHJ6*02 | 48.2 |
|  | BM |  |  | NA | 1.04 |
| **29** | LN | DLBCL | GCB | IGHV3-7*01IGHJ4*02 | 97.1 |
|  | BM |  |  | NA | 1.20 |

**Supp. Table 2A. DLBCL patients without morphologic evidence of lymphoma involvement in BM or flow abnormalities.** ; NA=not available. The percentage of reads with identical VDJ as the dominant clones in the matched DLBCL provide an upper limit of abundance of these minor clones in the BM.

| **Patient ID** | **Site** | **Diagnosis** | **Hans COO** | **MAJOR VJ** | **% reads (*DLBCL major VDJ*) in LN or BM** |
| --- | --- | --- | --- | --- | --- |
| **28** | Stomach, cardiac mass biopsy | DLBCL | GCB | IGHV3-30*03IGHJ6*02 | 75.24 |
|  | BM | Abnormal flow (CD5-CD10- B cells) |  | IGHV3-7*01 IGHJ3*02 | 0.05 |
| **32** | Testis | DLBCL | Non-GCB | IGHV4-30-4*04 IGHJ6*03 | 93.65 |
|  | BM | Abnormal flow (↓ k:l) |  | IGHV3-30*03 IGHJ5*02 | 0.00 |
| **41** | LN | DLBCL | Non-GCB | IGHV3-7*01 IGHJ4*02 | 95.74 |
|  | BM | Abnormal flow (CLL-like B-cells) |  | ND | 1.04 |
| **42** | LN | DLBCL | GCB | IGHV1-NL1*01 IGHJ6*02 | 59.86 |
|  | BM | Flow+ (CLL-like B-cells) |  | IGHV3-30*03 IGHJ4*02 | NA |
| **43** | LN | DLBCL | Non-GCB | IGHV2-5*10 IGHJ3*02 | 47.68 |
|  | BM | Abnormal flow (↑k:l) |  | IGHV3-64*01 IGHJ4*02 | 14.42 |

**Supp. Table 2B. DLBCL patients without morphologic evidence of lymphoma involvement in BM but with flow abnormalities.** Note the different major VJ rearrangements in 4 of the 5 cases, consistent with unrelated B-cell clones. ND= not detected; NA=not available

| **Patient ID** | **Site** | **Diagnosis** | **Hans COO** | **MAJOR VJ** | **% of total reads in LN or BM** |
| --- | --- | --- | --- | --- | --- |
| **15** | LN | DLBCL, EBV+ | Non-GCB | IGHV1-8*01 IGHJ6*02 | 86.9 |
|  | BM | DLBCL, EBV+ |  | IGHV1-8*01 IGHJ6*02 | 55 |
| **23** | LN | DLBCL +CD5+ LGBCL |  | IGHV4-34*01 IGHJ4*02 | 87.3 |
|  | BM | CD5+ LGBCL |  | IGHV4-34*01 IGHJ4*02 | 45.9 |
| **30** | LN | DLBCL +FL 3B | GCB | IGHV4-61*01 IGHJ6*03 | 92.53 |
|  | BM | DLBCL |  | IGHV4-61*01 IGHJ6*03 | 56.12 |
| **35** | LN | DLBCL + FL (Gr 3B and 1-2) | GCB | IGHV3-23*01 IGHJ6*02 | 93.18 |
|  | BM | DLBCL |  | IGHV3-23*01 IGHJ6*02 | 54.46 |
| **38** | LN | Plasmablastic lymphoma |  | IGHV4-39*01 IGHJ4*02 | 80.94 |
|  | BM | Plasmablastic lymphoma |  | IGHV4-39*01 IGHJ4*02 | 50.02 |
| **39** | Stomach | DLBCL | Non-GCB | IGHV1-69*13 IGHJ4*02 | 74.08 |
|  | BM1 | DLBCL |  | IGHV1-69*13 IGHJ4*02 | 84.43 |

**Supp. Table 2C. DLBCL patients with morphologic evidence of lymphoma involvement in BM.** LGBCL = low grade B-cell lymphoma
