# Supplementary material for: Exploring tumor clonal evolution in bone marrow of patients with diffuse large B-cell lymphoma by deep IGH sequencing and its potential relevance in relapse
Source: Blood Cancer J. 2019 Aug 21;9(9):69. doi: 10.1038/s41408-019-0229-1 (PMC6704167; doi:10.1038/s41408-019-0229-1)
Supplement: Supplementary file 3 — Supplementary table 3 [file 41408_2019_229_MOESM3_ESM.docx]

| **Patient ID** | **Types of clones** | **Clinical status** |
| --- | --- | --- |
| **4** | undetectable | 0 |
| **5** | undetectable | 0 |
| **9** | undetectable | 0 |
| **10** | I | 0 |
| **12** | I | 0 |
| **19** | DA & I | R |
| **20** | I | 0 |
| **21** | DA & I | R |
| **29-1** | DA | R |
| **41** | DA | R |
| **42** | DA | 0 |
| **43** | DA & I | 0 |

**Supp Table 3. Types of minor clones (DA or I) detected in staging BM of patients with DLBCL and correlation with subsequent DLBCL relapse.** 0, No relapse; R, relapse; DA, Divergent/ancestral clones; I, identical clones
