# Supplementary material for: Exploring tumor clonal evolution in bone marrow of patients with diffuse large B-cell lymphoma by deep IGH sequencing and its potential relevance in relapse
Source: Blood Cancer J. 2019 Aug 21;9(9):69. doi: 10.1038/s41408-019-0229-1 (PMC6704167; doi:10.1038/s41408-019-0229-1)

Supplementary Figure 1.

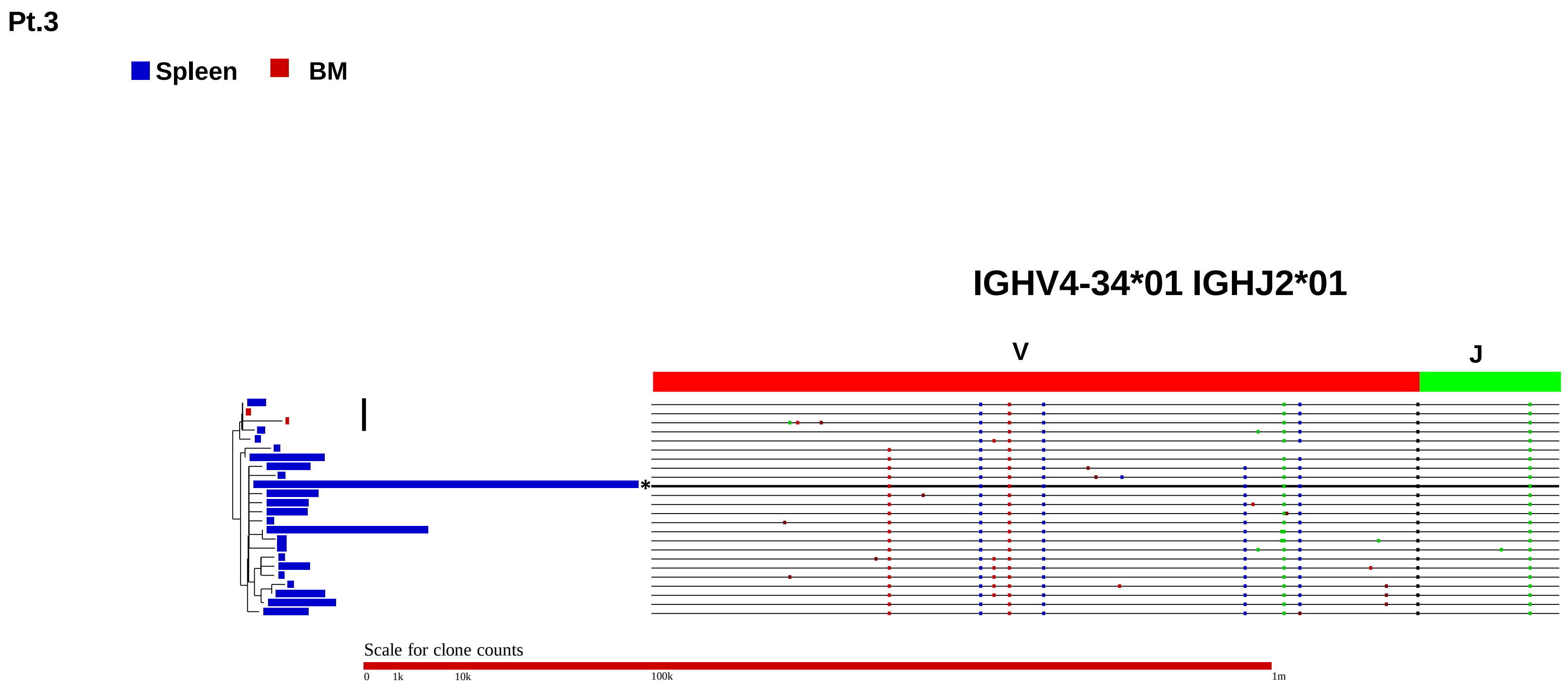

Pt. 8

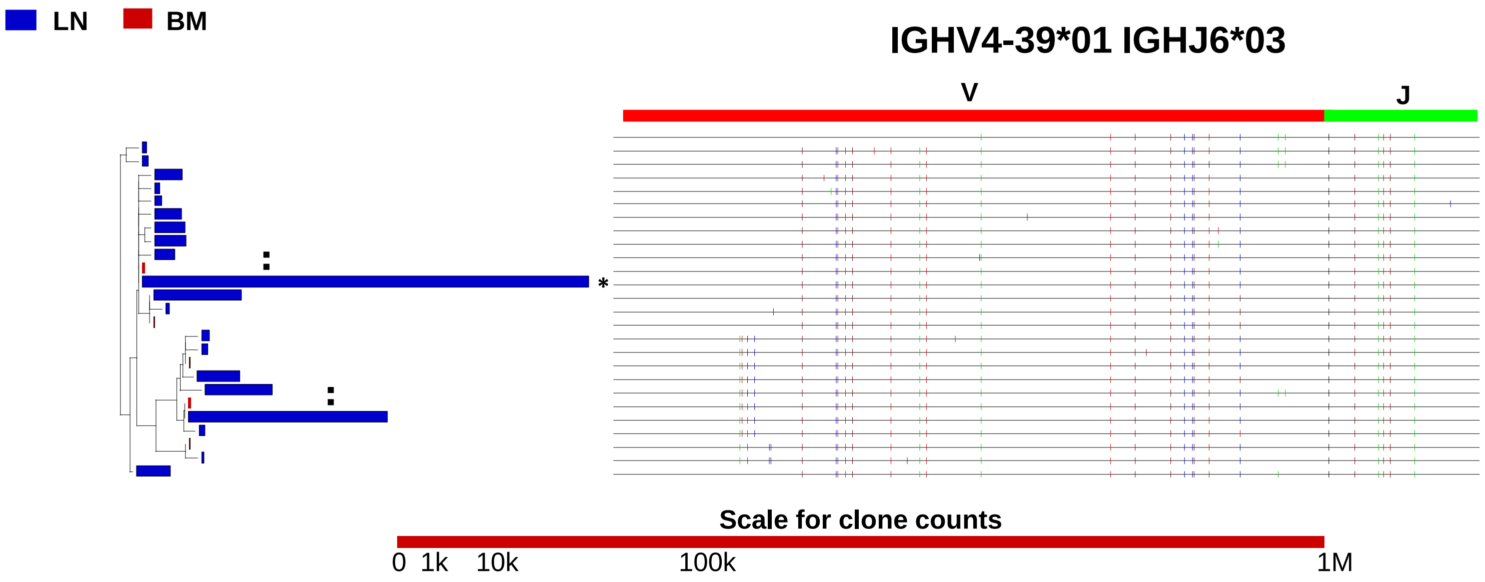

LN
BM
IGHV4-39*01 IGHJ6*03
V
J
*
Scale for clone counts
0
1k
10k
100k
1M

Pt. 10

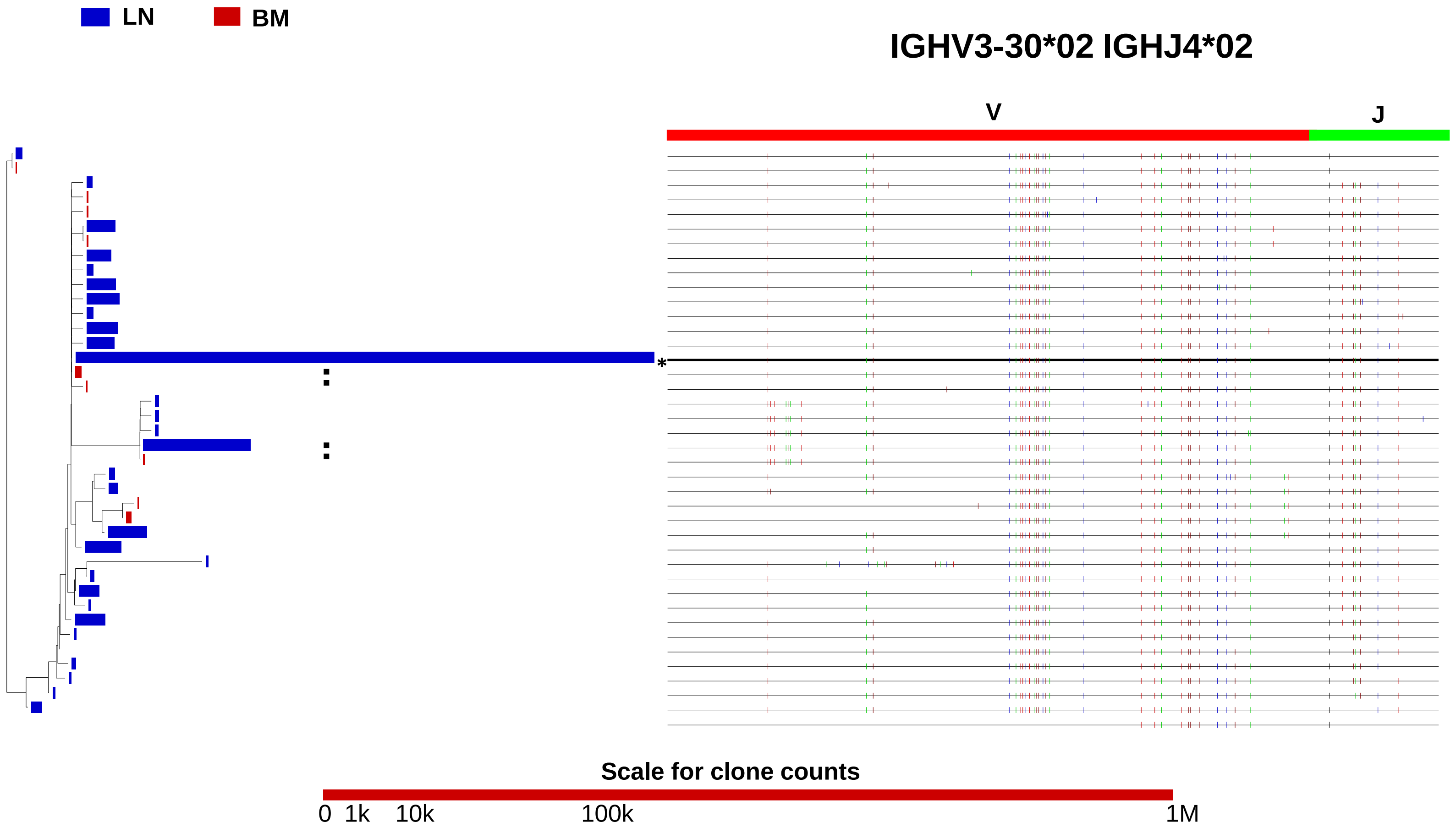
LN
BM
IGHV3-30*02 IGHJ4*02
V
J
*
Scale for clone counts
0
1k
10k
100k
1M

Pt. 12

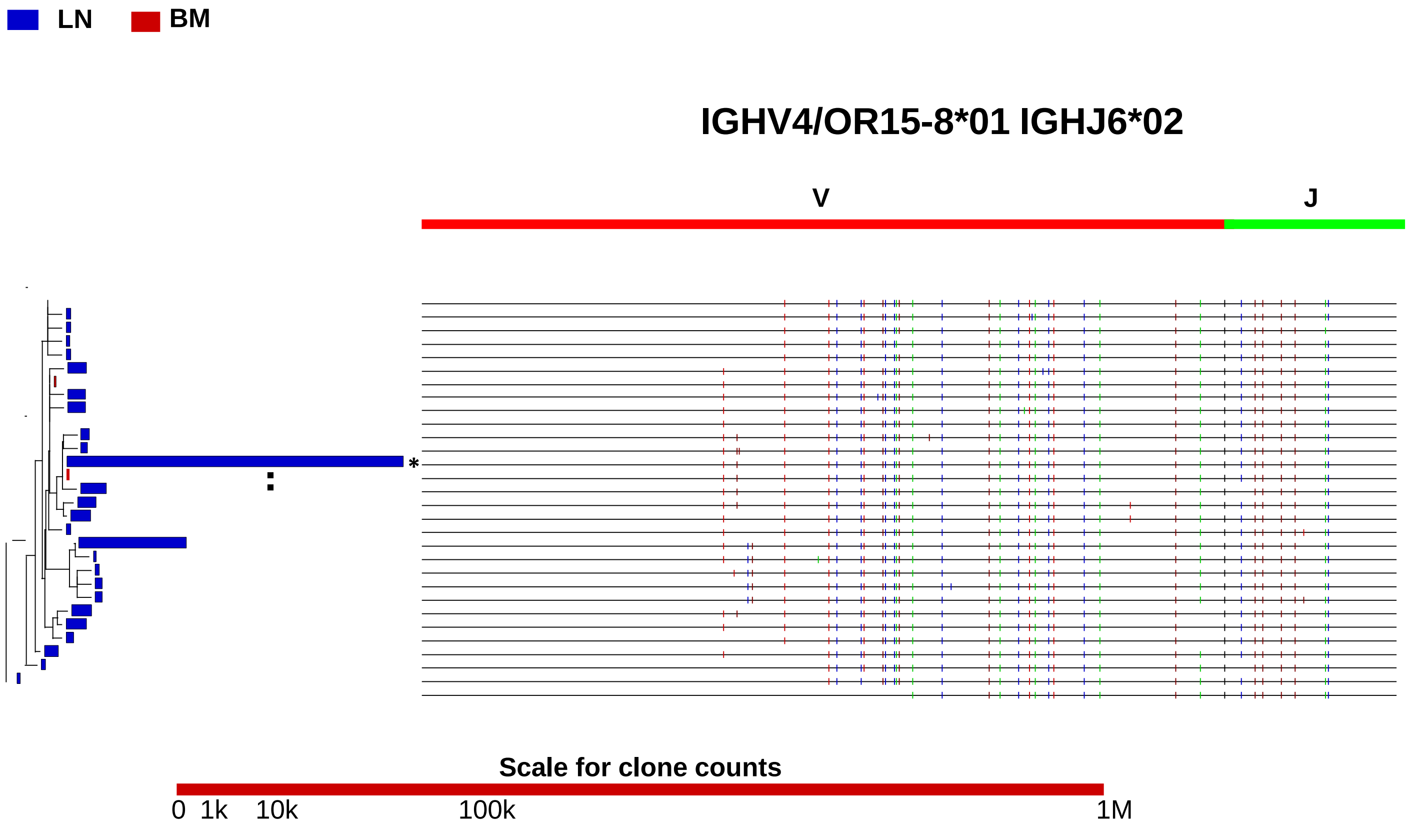
LN
BM
IGHV4/OR15-8*01 IGHJ6*02
V
J
*
Scale for clone counts
0
1k
10k
100k
1M

Pt. 14

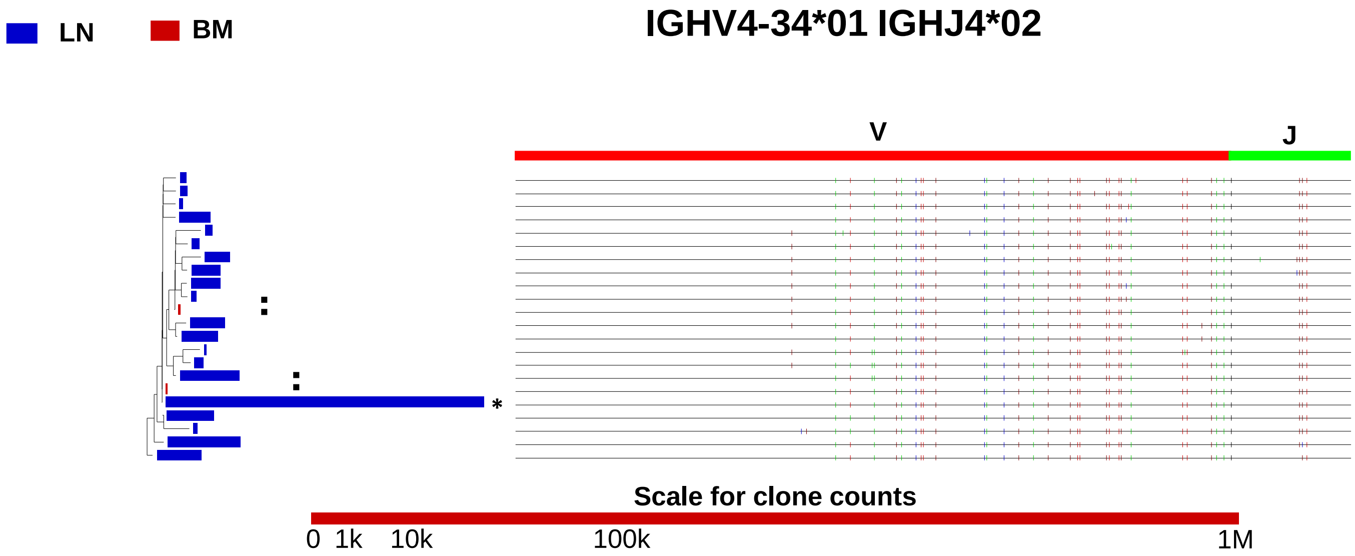
LN
BM
IGHV4-34*01 IGHJ4*02
V
J
*
Scale for clone counts
0 1k 10k 100k 1M

Pt. 17

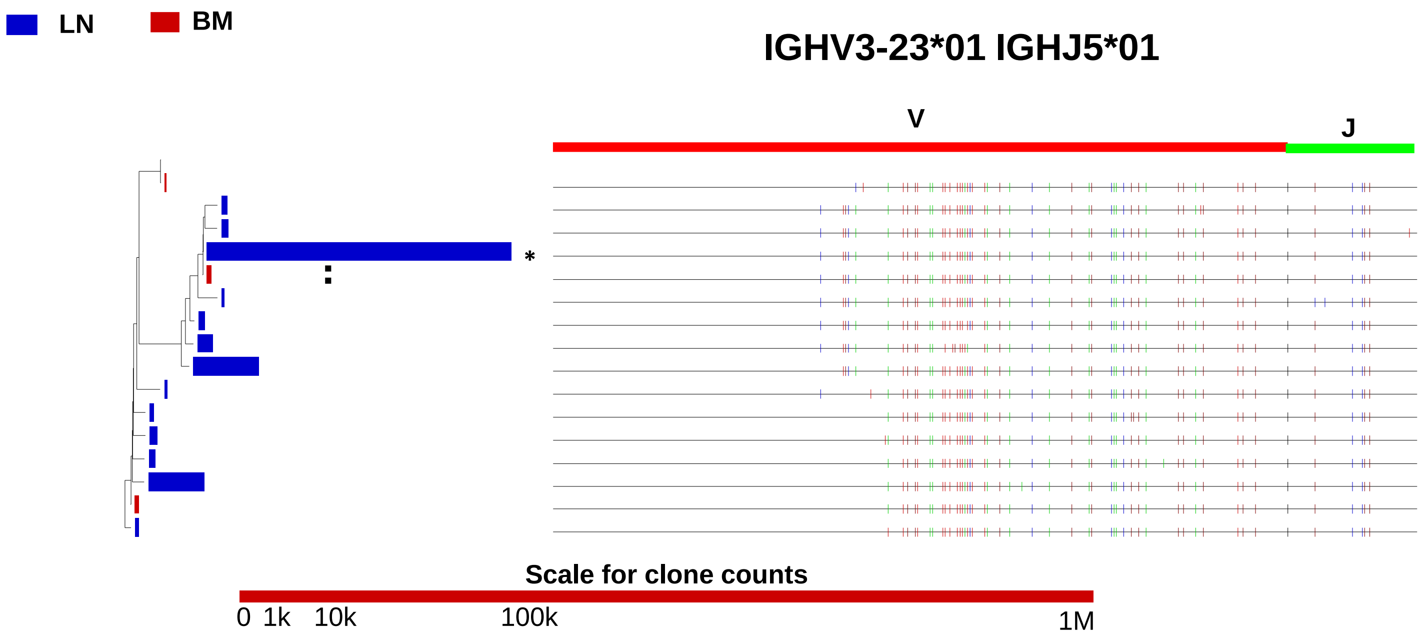
LN
BM
IGHV3-23*01 IGHJ5*01
V
J
*
Scale for clone counts
0
1k
10k
100k
1M

Pt. 18

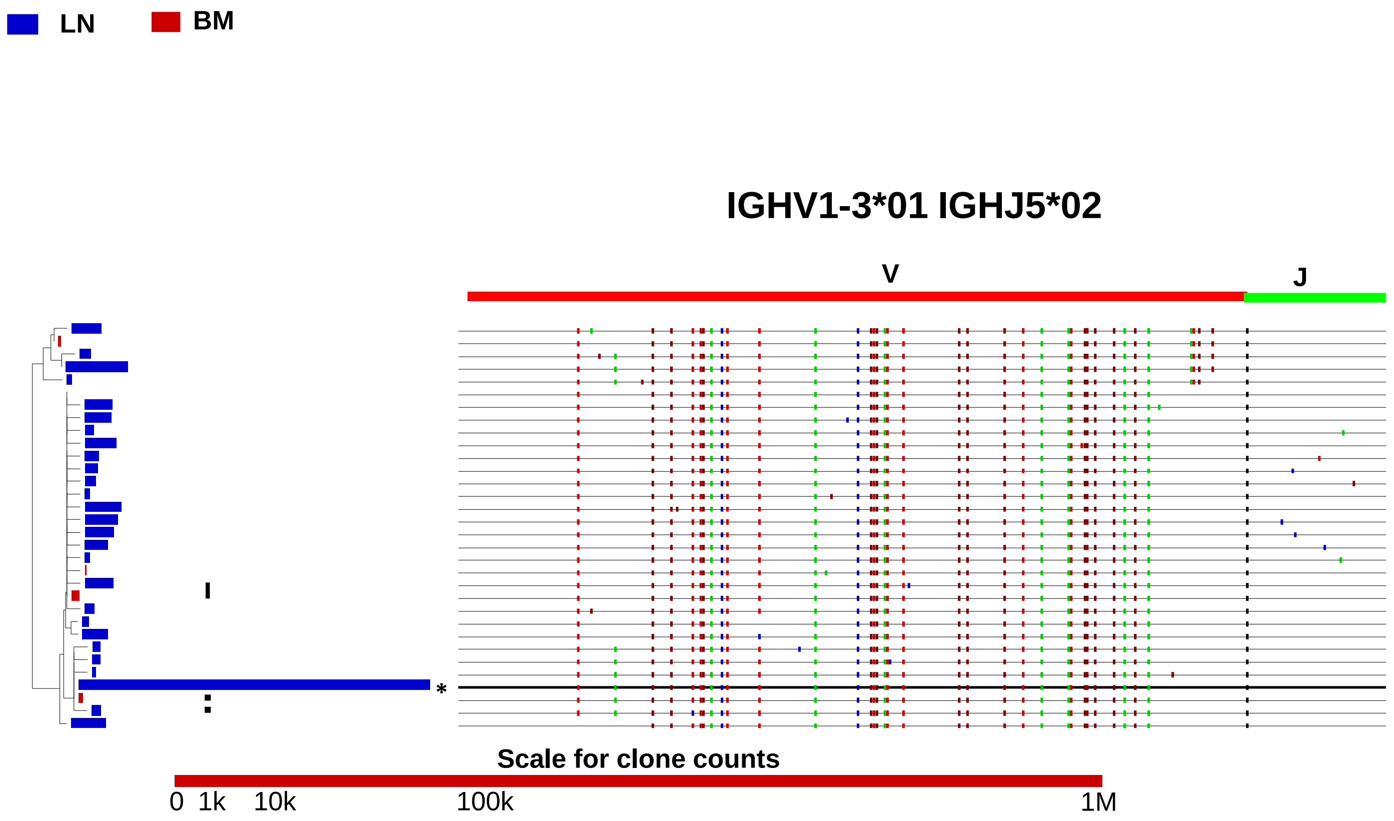
LN
BM
IGHV1-3*01 IGHJ5*02
V
J
*
Scale for clone counts
0
1k
10k
100k
1M

Pt. 19

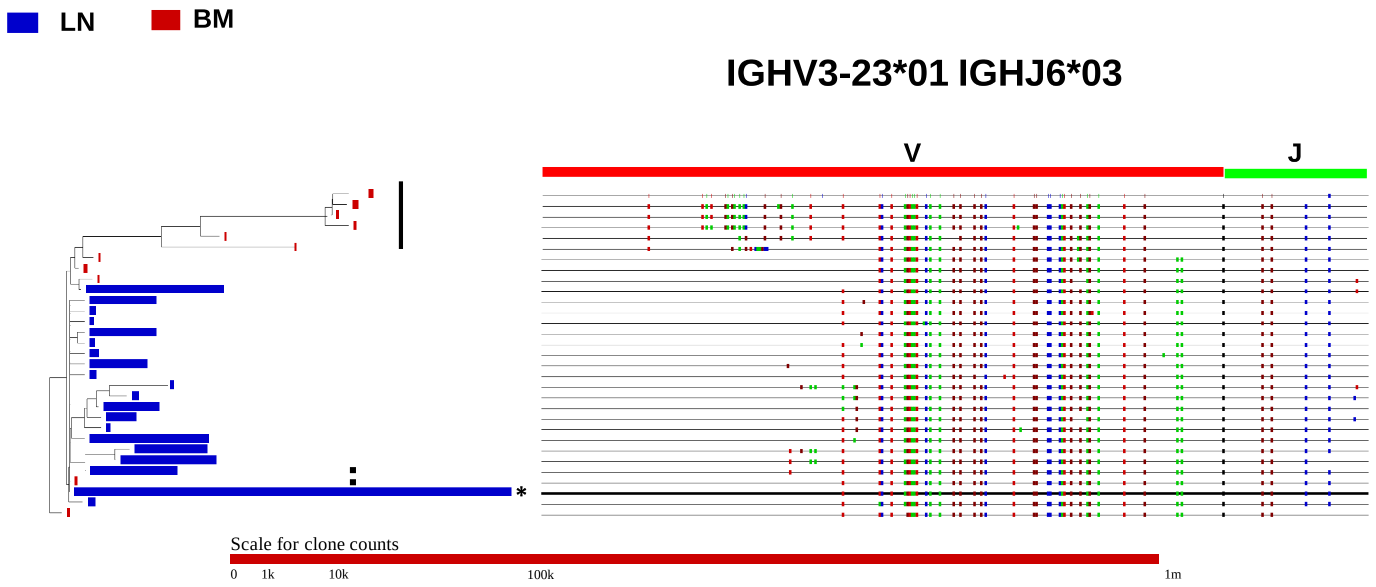
LN
BM
IGHV3-23*01 IGHJ6*03
V
J
*
Scale for clone counts
0
1k
10k
100k
1m

Pt. 20

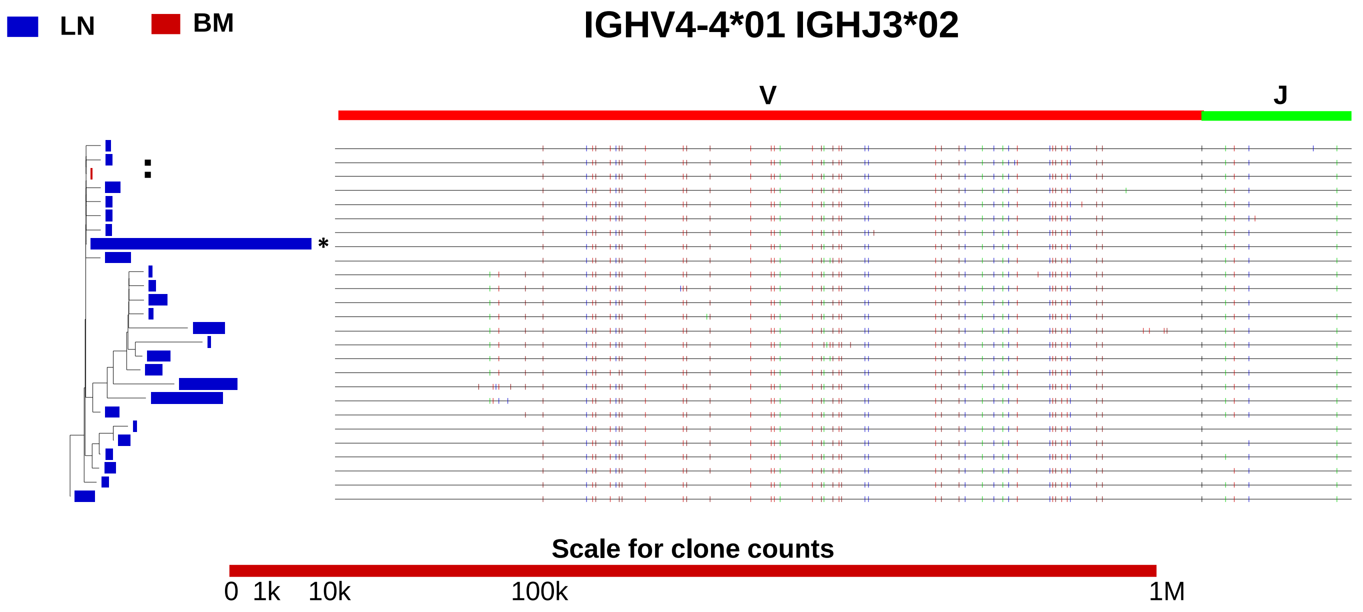

LN
BM
IGHV4-4*01 IGHJ3*02
V
J
*
Scale for clone counts
0
1k
10k
100k
1M

Pt. 21

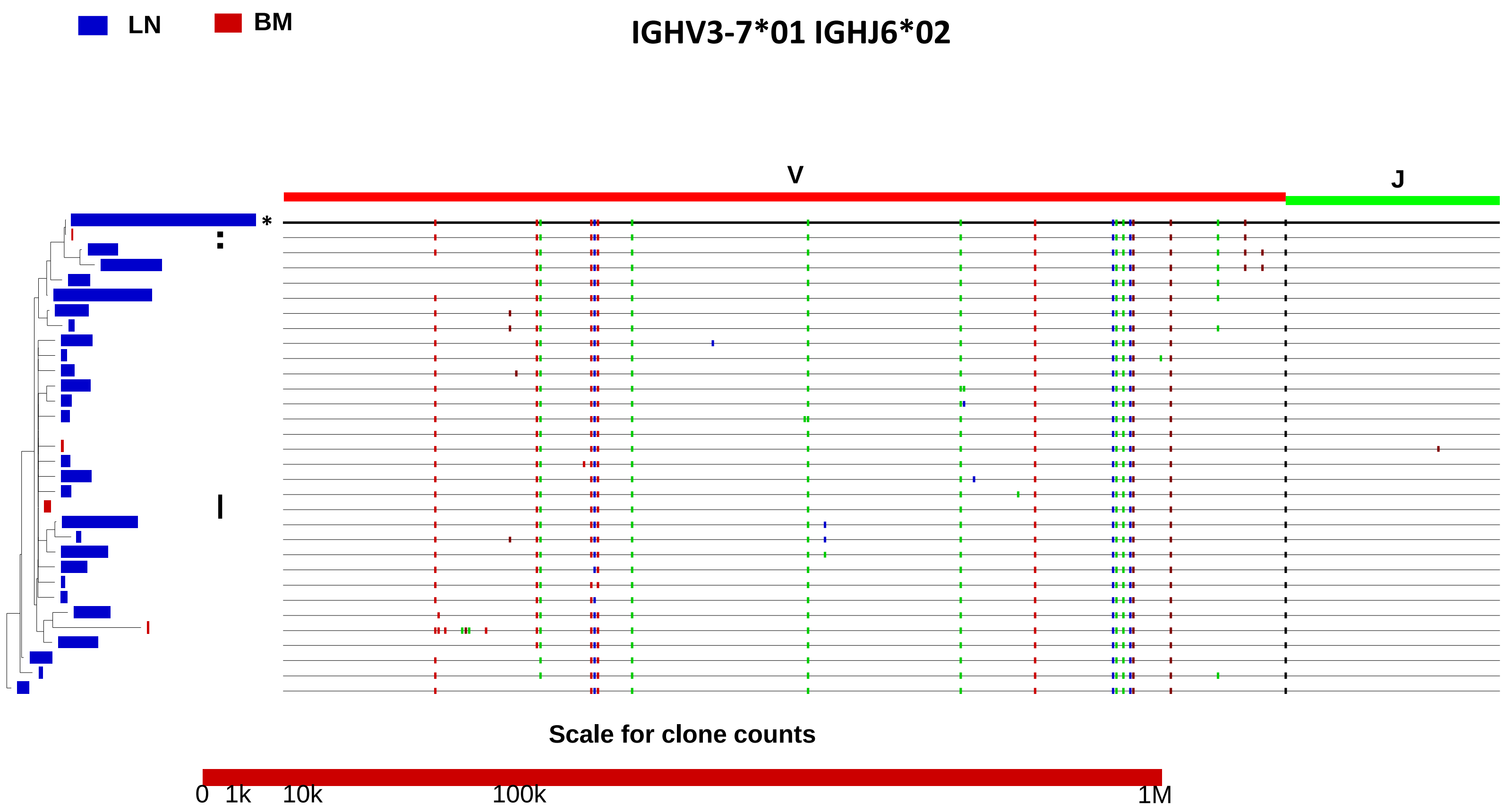

LN
BM
IGHV3-7*01 IGHJ6*02
V
J
*
Scale for clone counts
0
1k
10k
100k
1M

Pt. 29-1

v

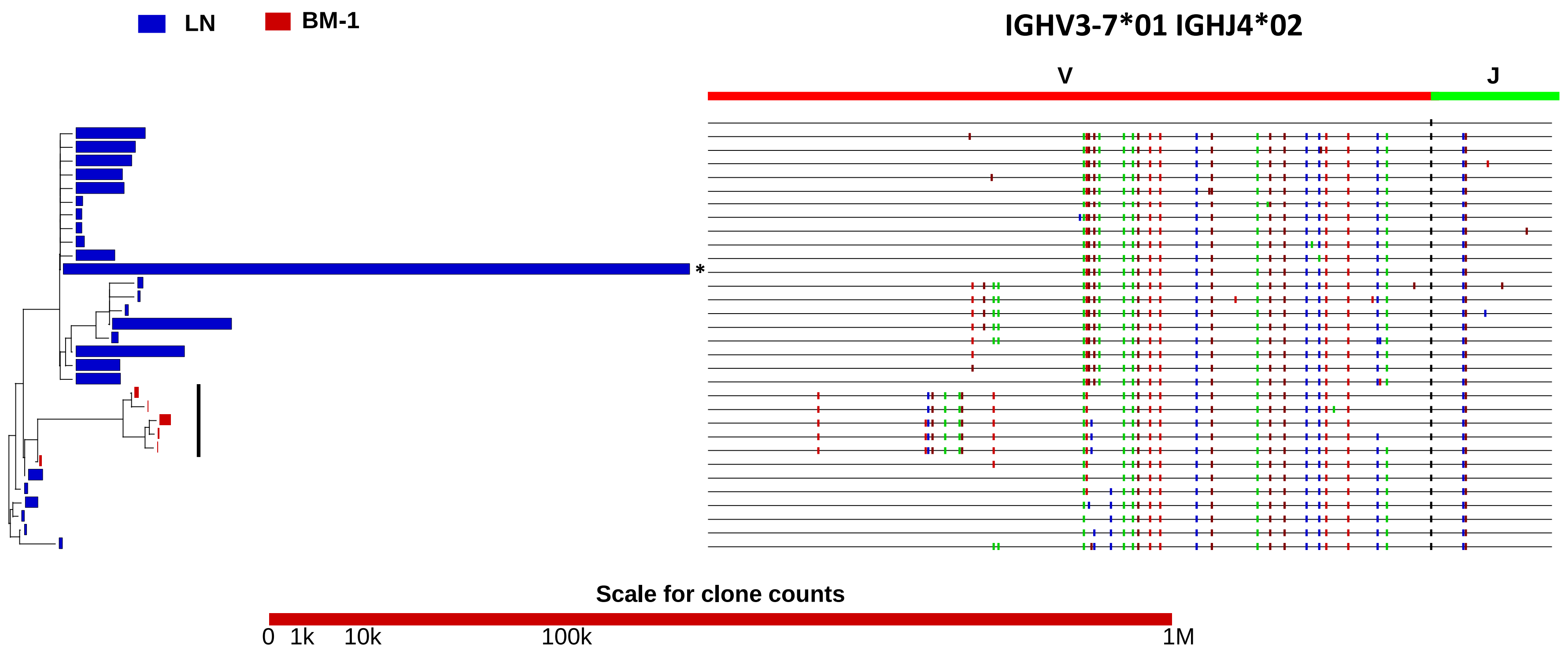
LN
BM-1
IGHV3-7*01 IGHJ4*02
V
J
*
Scale for clone counts
0 1k 10k 100k 1M

Pt. 29-2

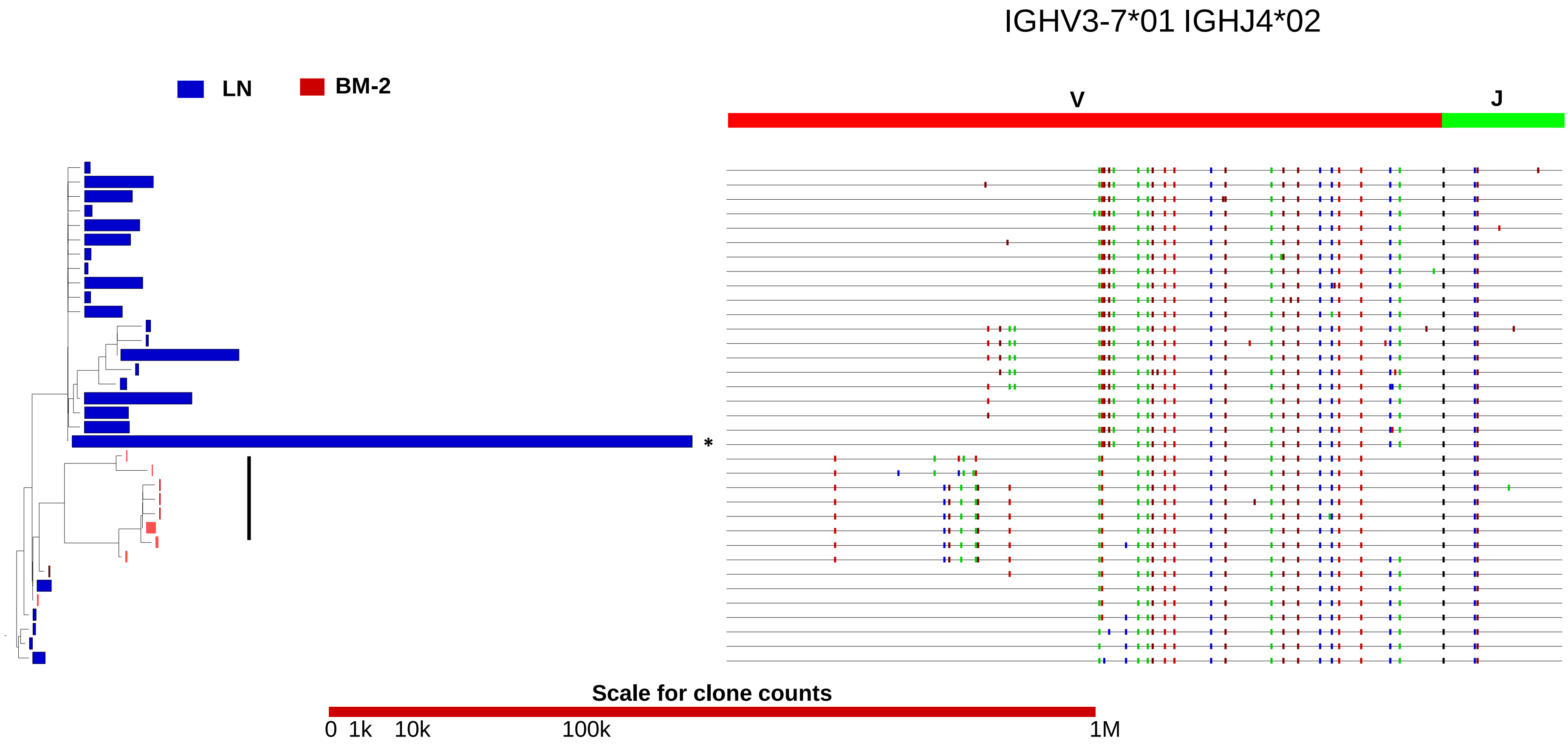

IGHV3-7*01 IGHJ4*02
LN
BM-2
V
J
*
Scale for clone counts
0 1k 10k 100k 1M

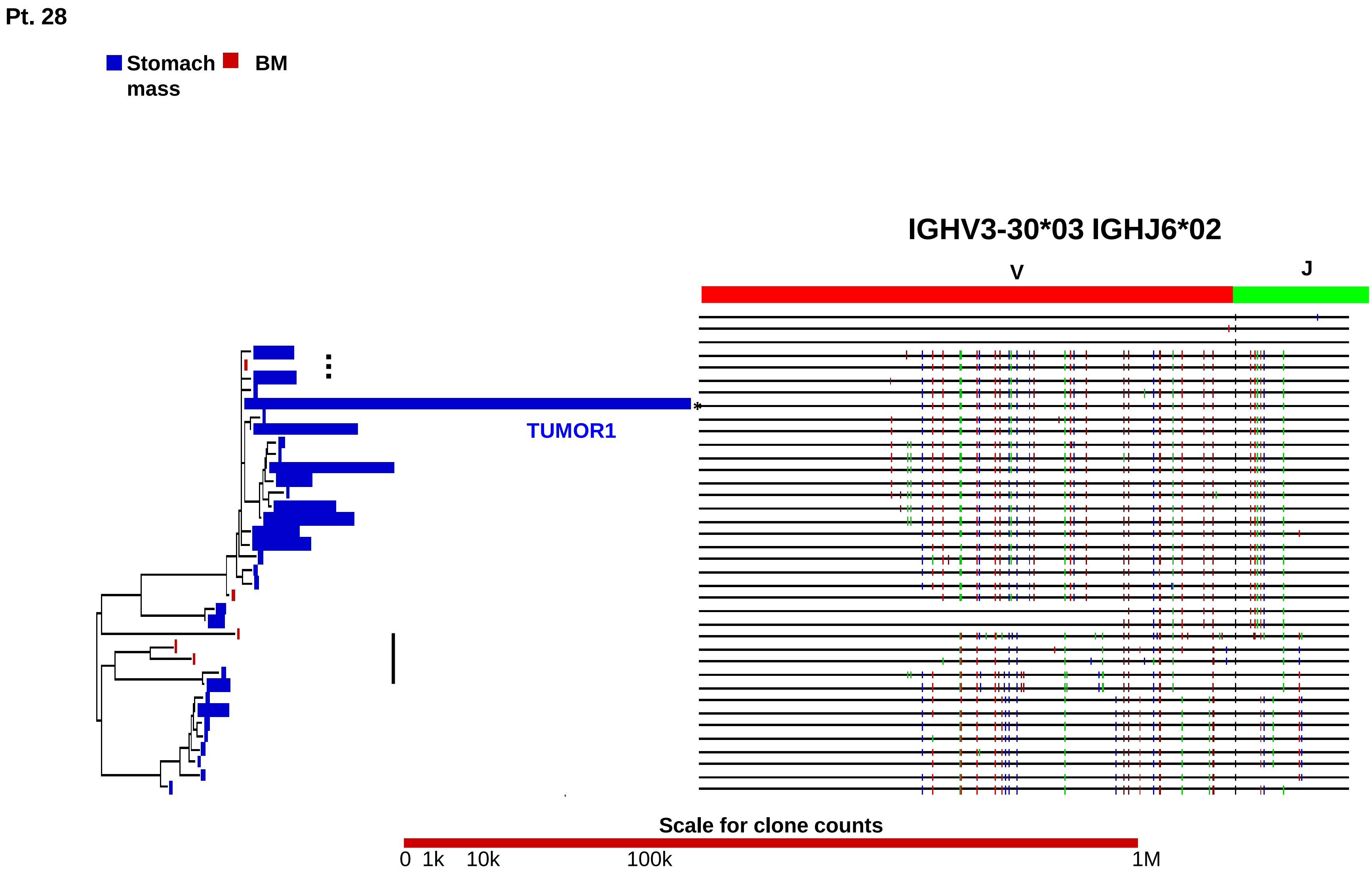

Pt. 28
Stomach mass
BM
IGHV3-30*03 IGHJ6*02
V
J
TUMOR1
Scale for clone counts
0
1k
10k
100k
1M

Pt. 32

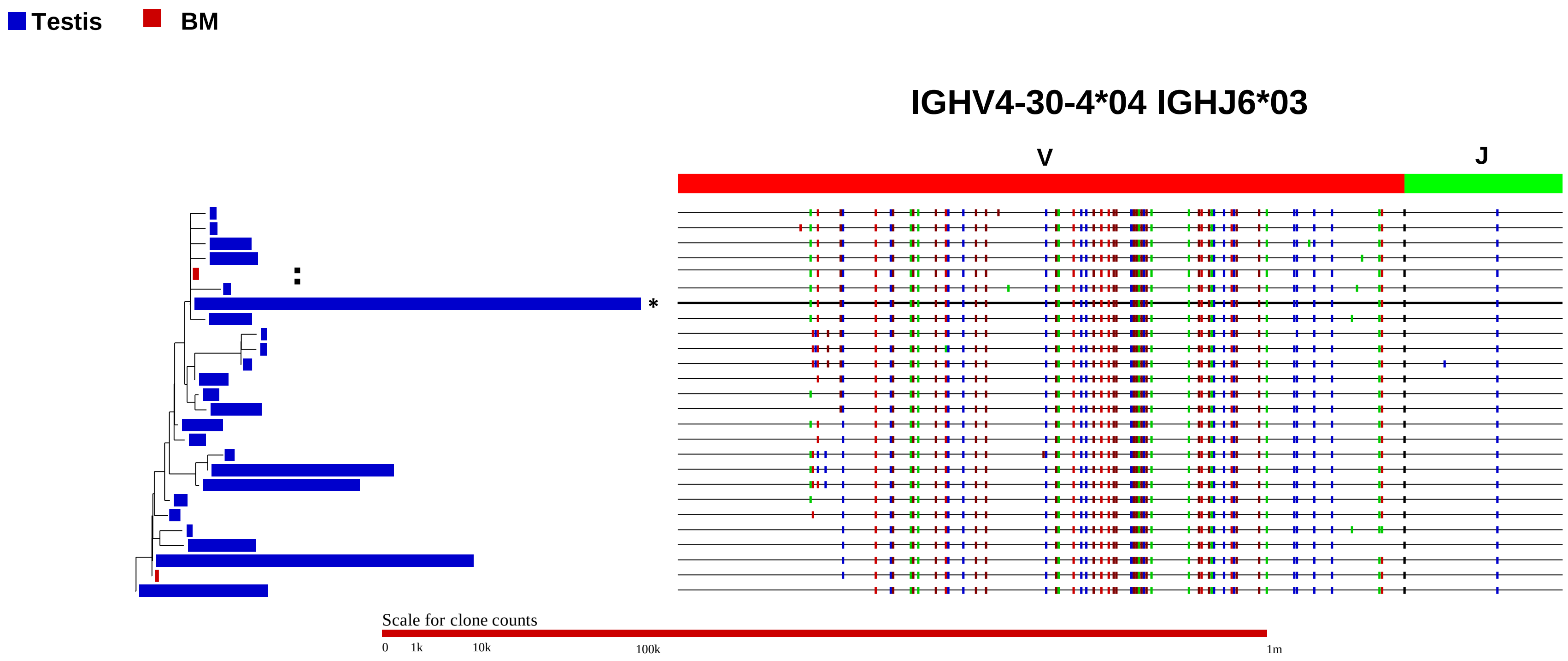
Testis
BM
IGHV4-30-4*04 IGHJ6*03
V
J
*
Scale for clone counts
0
1k
10k
100k
1m

Pt. 41

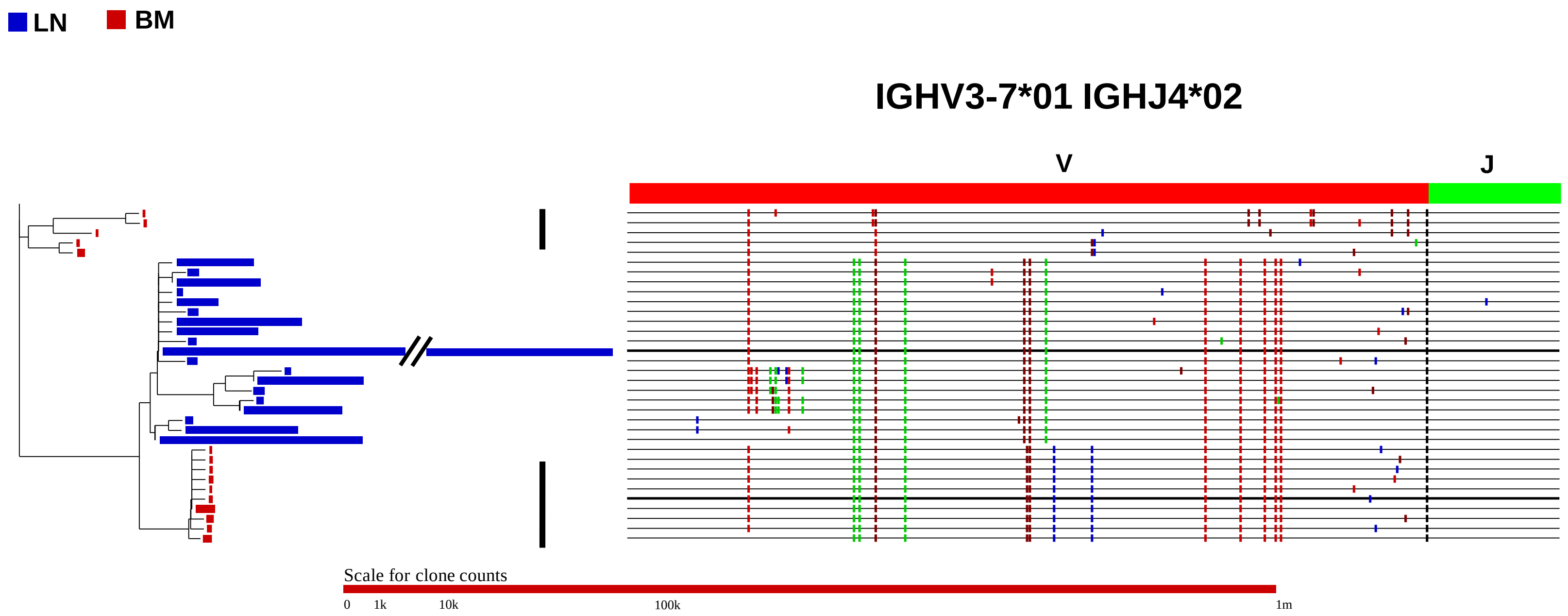
LN
BM
IGHV3-7*01 IGHJ4*02
V
J
Scale for clone counts
0
1k
10k
100k
1m

Pt. 42

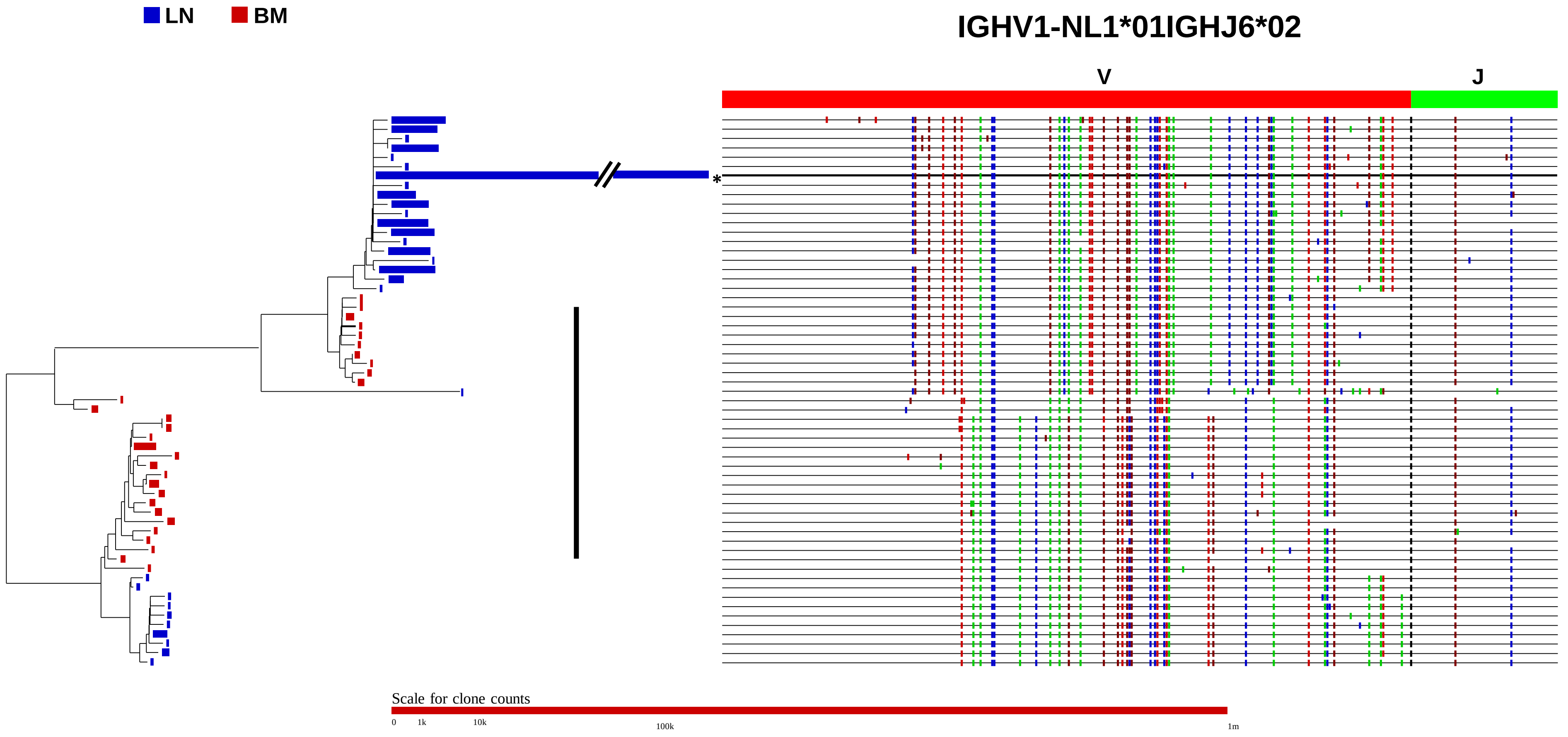
LN
BM
IGHV1-NL1*01IGHJ6*02
V
J
*
Scale for clone counts
0
1k
10k
100k
1m

Pt. 43

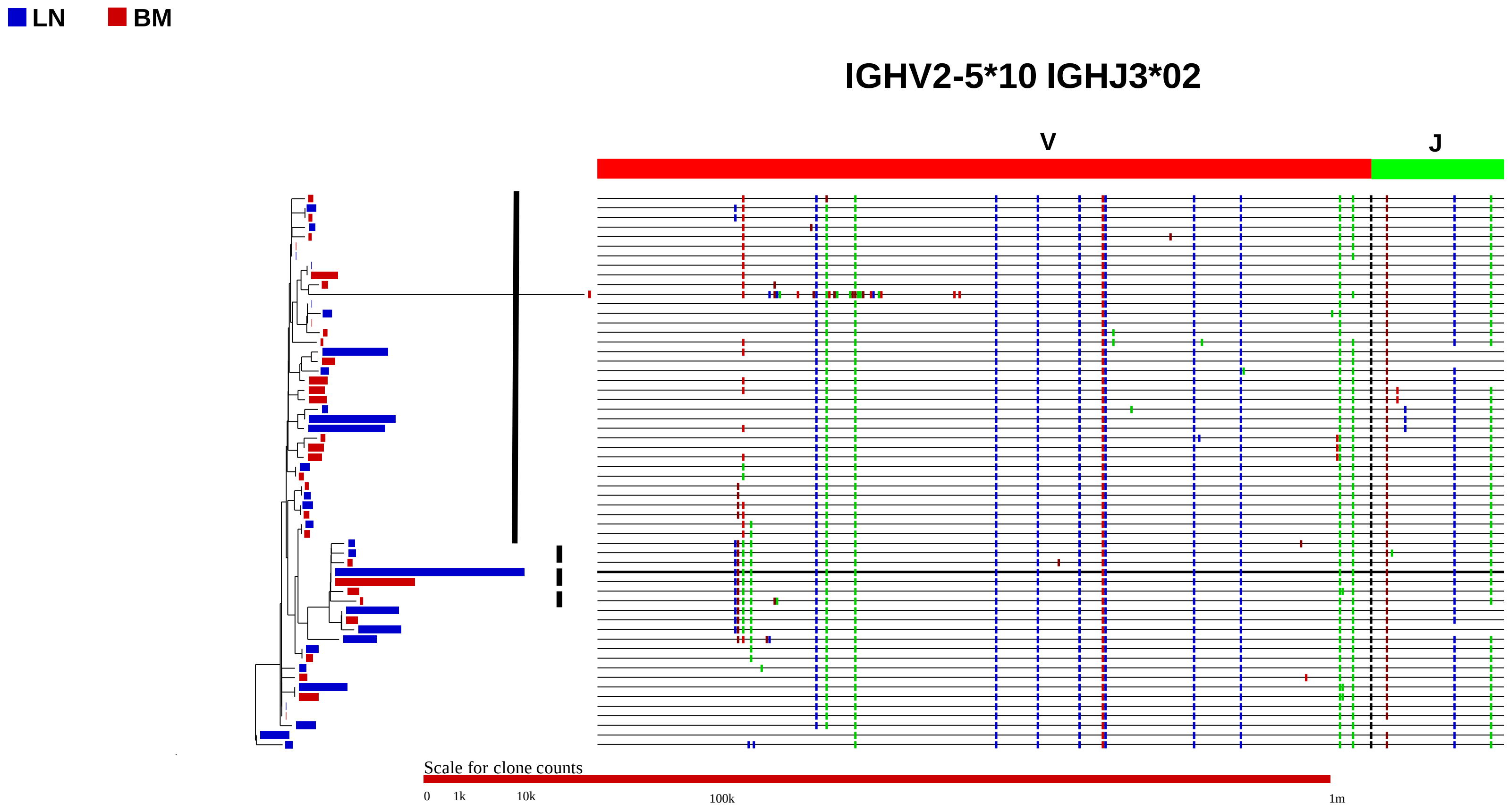
LN
BM
IGHV2-5*10 IGHJ3*02
V
J
Scale for clone counts
0
1k
10k
100k
1m

Supplementary Figure 2

Pt. 15

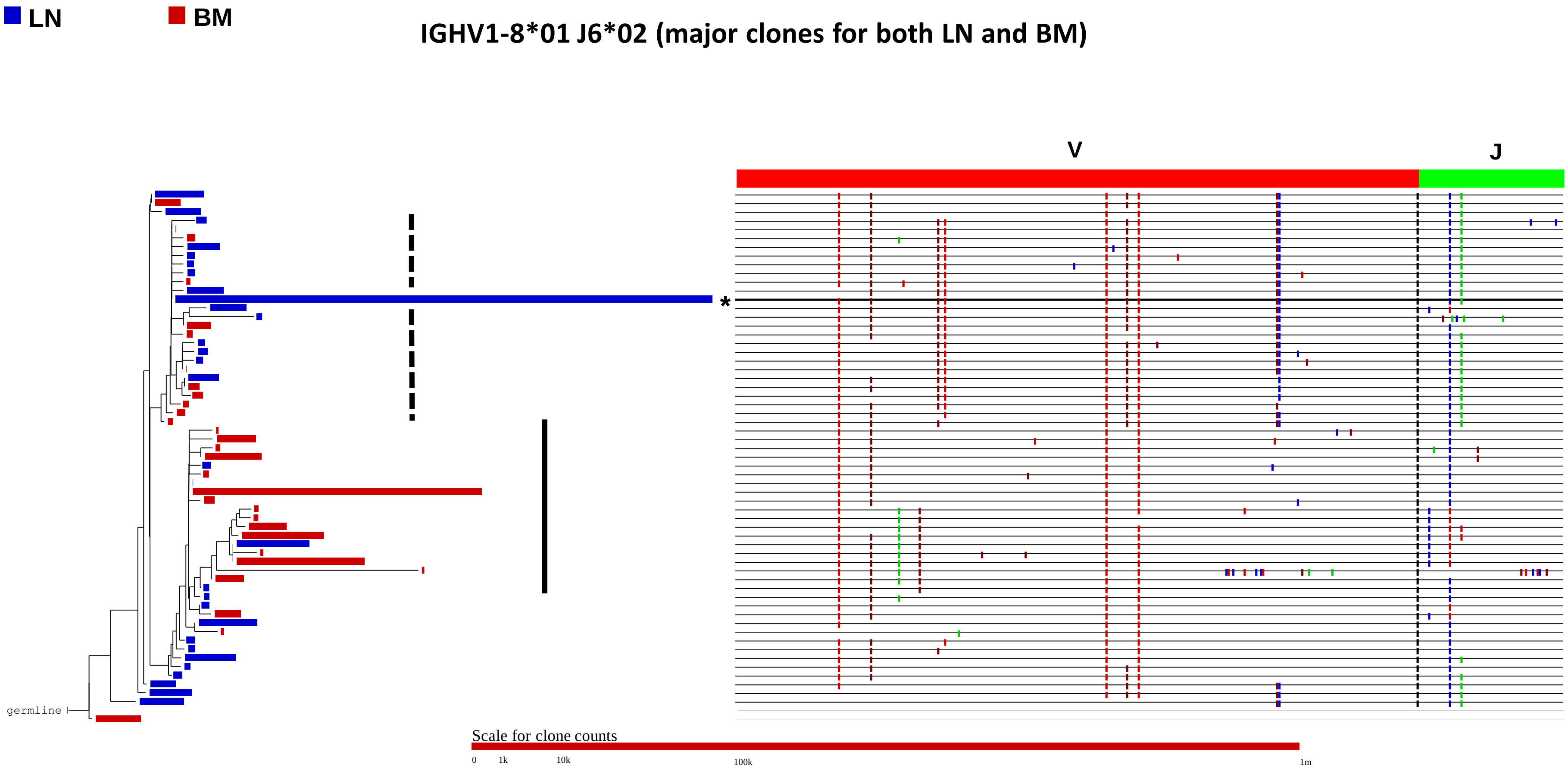

Pt. 23

LN BM

IGHV4-34*01 IGHJ4*02 (major clones in LN and BM)

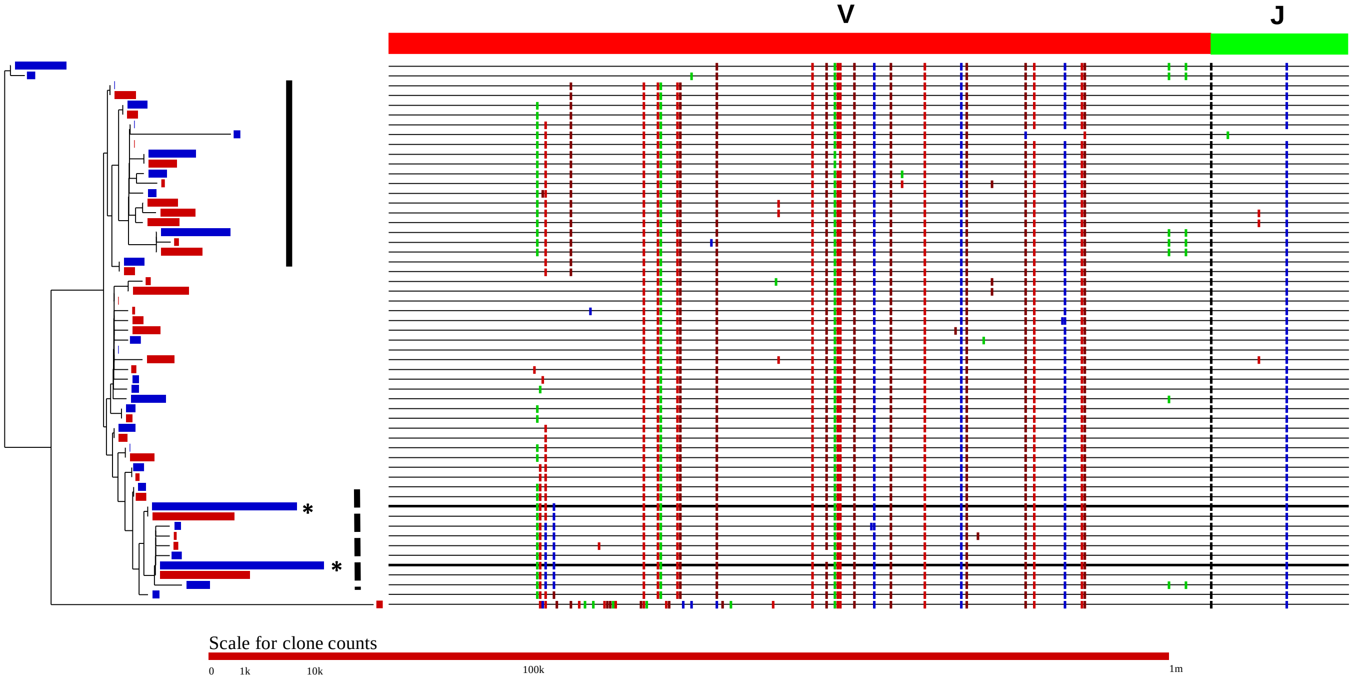

Pt. 30

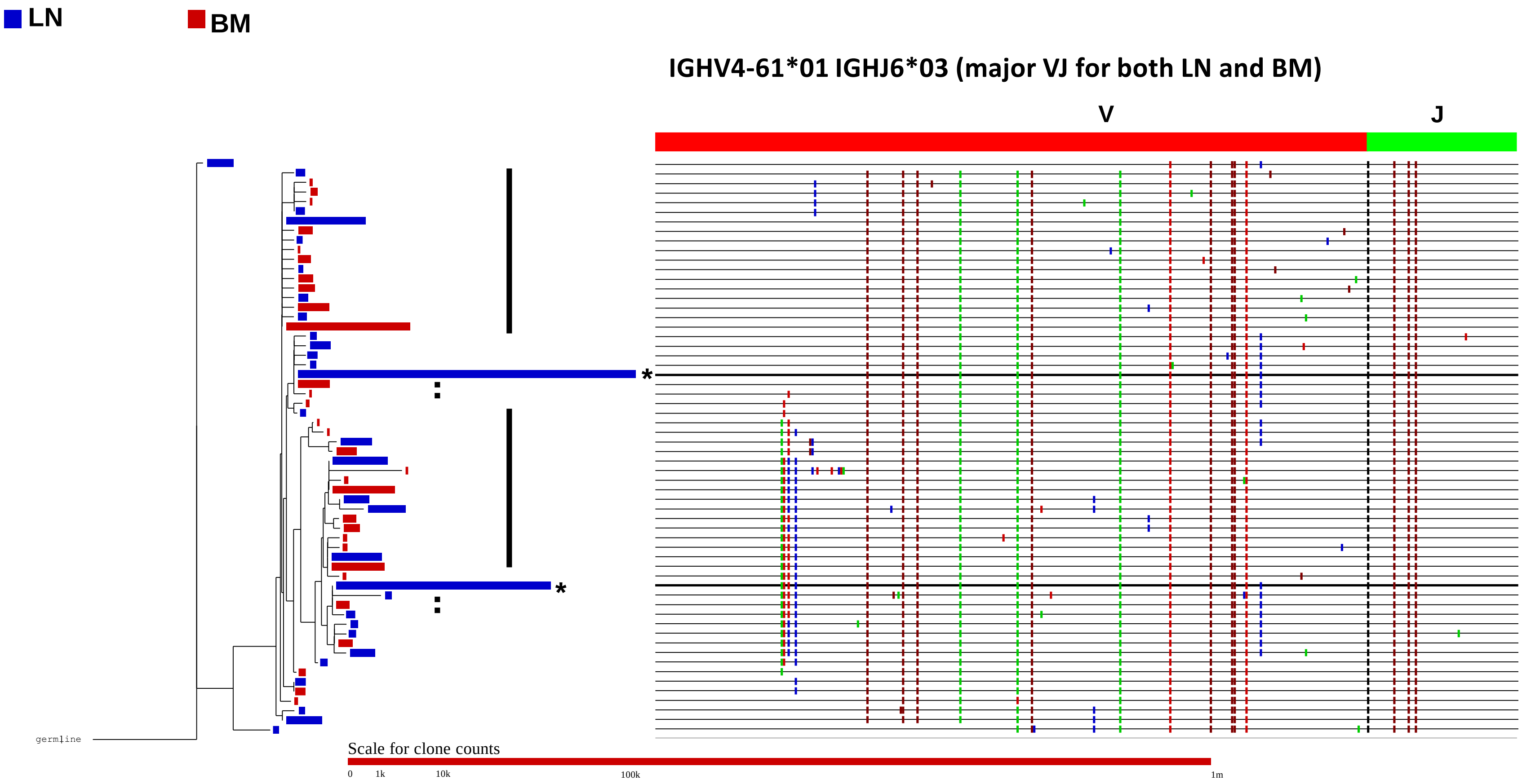
LN
BM
IGHV4-61*01 IGHJ6*03 (major VJ for both LN and BM)
V
J
germline
Scale for clone counts
0
1k
10k
100k
1m

Pt. 35

LN BM

IGHV3-23*01 IGHJ6*02 (major clones for both LN and BM)

Dominant VDJ clone i
LN: IGHV3-23*01 IGH
BM: IGHV3-23*01 IGI

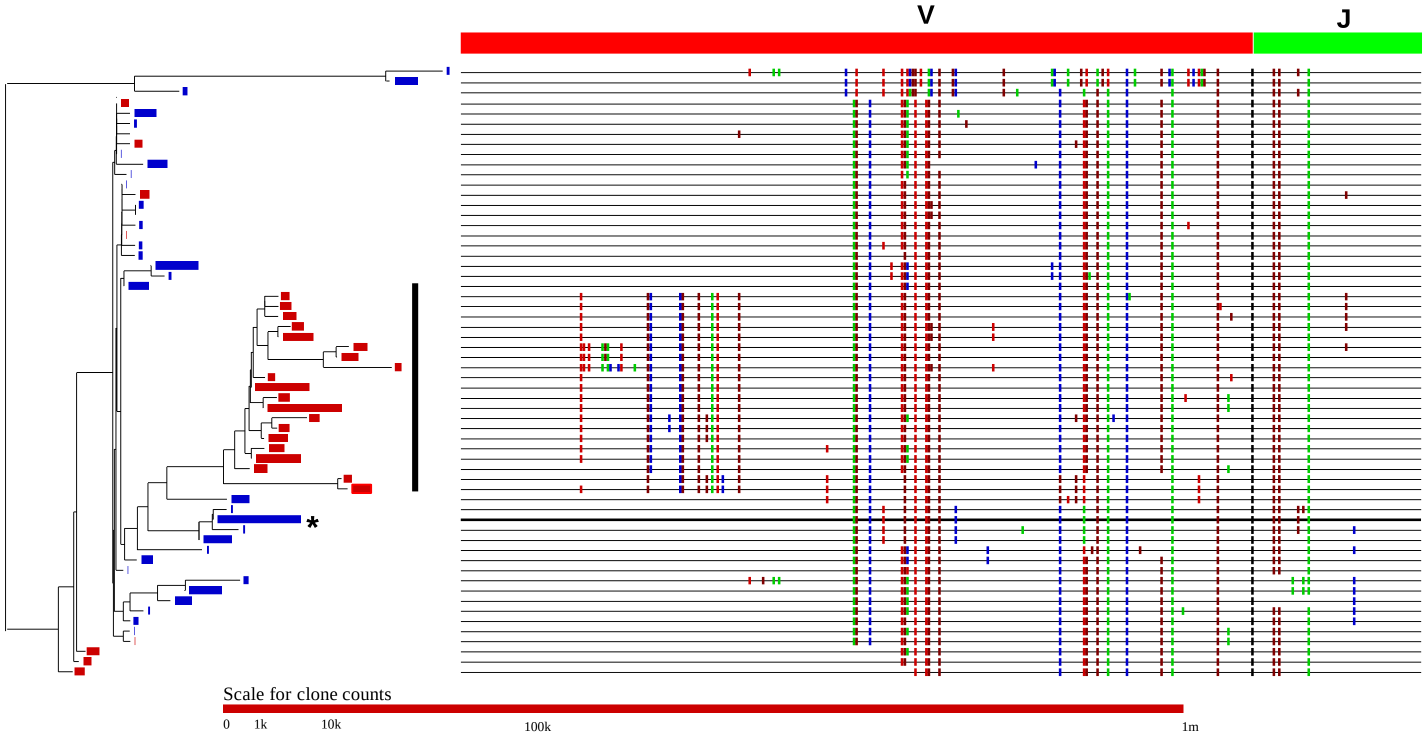

D-type clones in lymph node

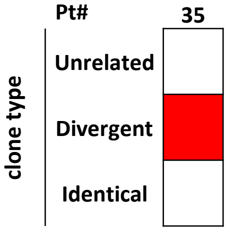

Pt. 38

IGHV4-39*01 J4*02 (major clones for both LN and BM)

LN BM

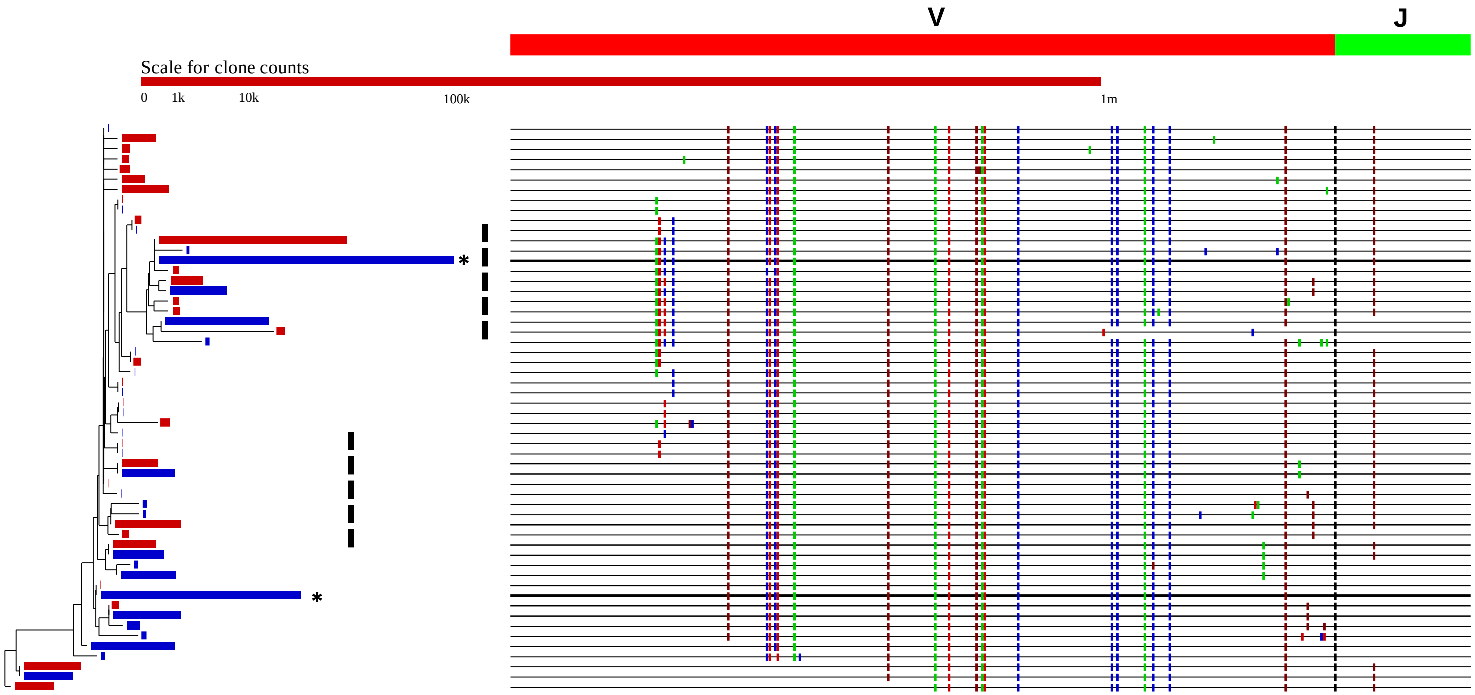

Pt. 39

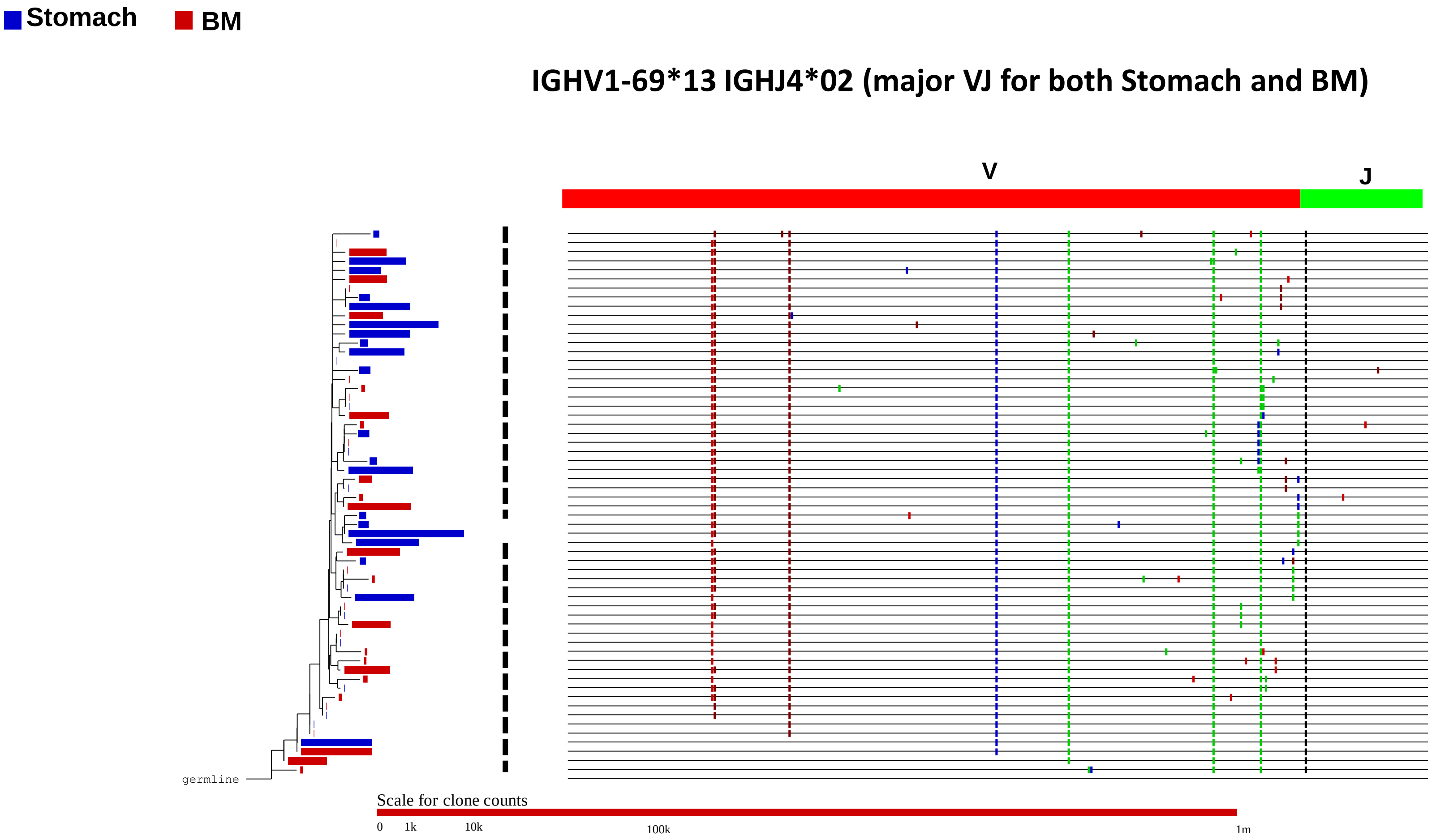

Stomach
BM
IGHV1-69*13 IGHJ4*02 (major VJ for both Stomach and BM)
V
J
germline
Scale for clone counts
0
1k
10k
100k
1m

Supplementary Figure 3

A.

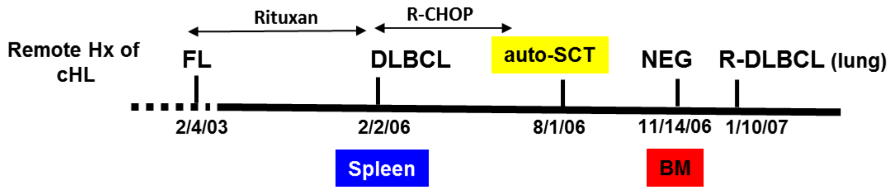

B.

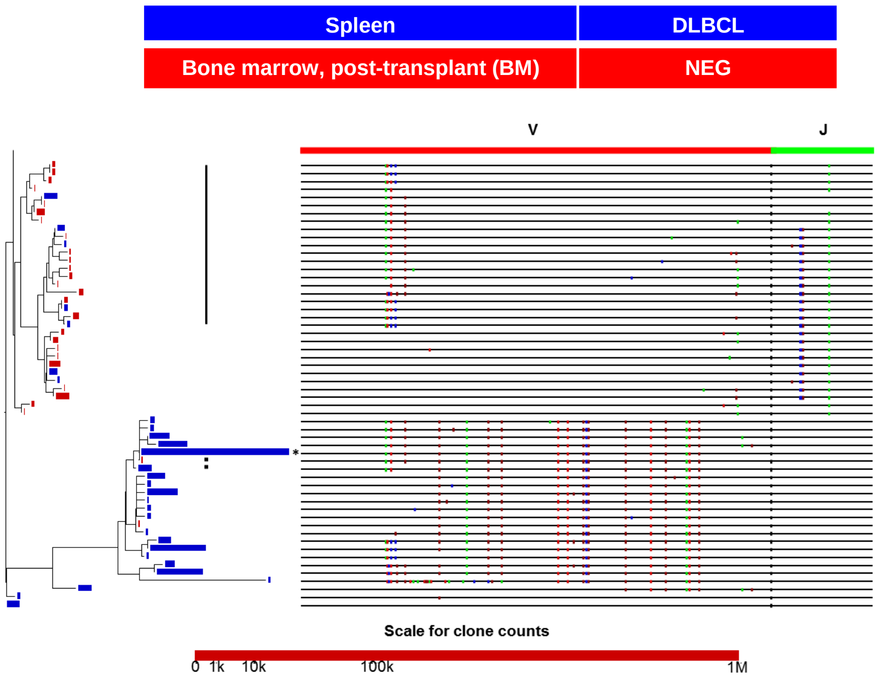

IGHV4-34*01 IGHJ6*03

Supplementary Figure 4

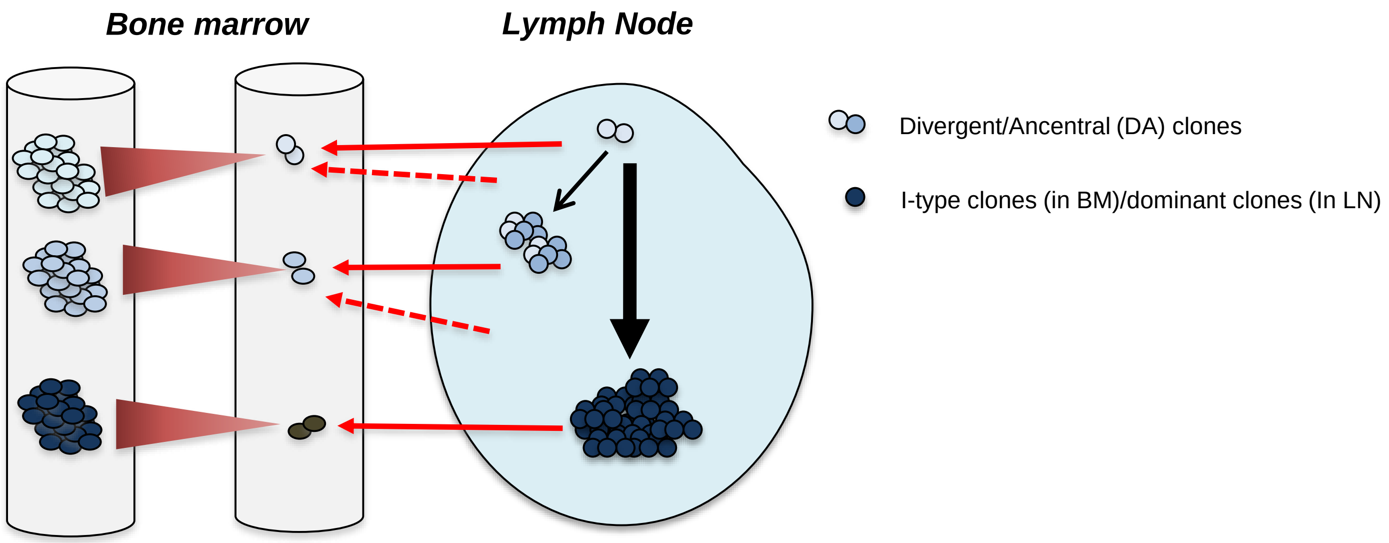

Supplement: Supplementary file 4 — Supplementary figures [file 41408_2019_229_MOESM4_ESM.pdf]
