# Supplementary material for: Exploring tumor clonal evolution in bone marrow of patients with diffuse large B-cell lymphoma by deep IGH sequencing and its potential relevance in relapse
Source: Blood Cancer J. 2019 Aug 21;9(9):69. doi: 10.1038/s41408-019-0229-1 (PMC6704167; doi:10.1038/s41408-019-0229-1)
Supplement: Supplementary file 5 — Supplementary file and Figure legends [file 41408_2019_229_MOESM5_ESM.pdf]

Supplementary File and Figure Legends

1. Materials and Methods.

Sample selections

Diagnosis and relapse DLBCL cases, and bone marrow specimen were selected from a search of the database of the Department of Pathology and Laboratory Medicine at Weill Cornell Medicine. Information regarding clinical history and presentation, therapy and follow-up was obtained from electronic clinical records. All patients provided written consent for use of tissues samples for research, in accordance with the Declaration of Helsinki regulations of the protocols approved by the Institutional Review Board of Weill Cornell Medical College, New York, USA (IRB # 0107004999).

DNA extraction

DNA was extracted from the frozen tissue sections using the salting out method. DNA was extracted from BM frozen cells or cytogenetics pellets using the Qiagen DNeasy Blood & Tissue Kit (Valencia, CA) according to instruction manual. In brief, cell pellets were washed three times in 1 x PBS to remove the fixation buffer, then digested with 20 μl of Proteinase K inhibitor followed by 200 μl buffer AL at 56°C for 10min. After digestion, 200 μl of 100% ethanol was added to the cells, and DNA was bind to the column at 8000 rpm for 1min. Flow through was discarded, and column was washed once with buffer AW1, followed by twice wash with buffer AW2. DNA was finally eluted in AE buffer at room temperature. DNA integrity of each sample was checked on a 0.8% agarose gel prior to the PCR amplification.

PCR amplification of Fr1 or Fr2 IGH VDJ rearranged region

IgVHRF1 was PCR amplified using Mix 2 of the Somatic Hypermutation assay v2.0 assay (Cat. No. 5-101-0030) from InvivoScribe Technologies (San Diego, CA) according to the instruction manual. This Master mix targets sequences between the framework 1 (FR1) and joining (J) regions. The resulting amplicons include a portion of the FR1 region to the downstream J region. For each PCR reaction, 200 ng of input DNA alongside 45 μl of Mix 2 master mix, 0.25 μl AmpliTaq Gold DNA polymerase, and topped up to 50 μl total reaction with appropriate amount of ddH2O. FR1 fragment was then PCR amplified using the thermocycler program at 95°C for 7 minutes, 95°C for 45 seconds, 60°C for 45 seconds, 72°C for 90 seconds, then go to step 2, repeat 34 more times, 72°C for 10 minutes, 15°C forever. Fr1 PCR fragment were resolved on 2% TAE agarose gel alongside a panel of quality controls from the kit, which are negative control also known as polyclonal control (IVS-0000), positive control or clonal control (IVS-0013), no template control and specimen size control ladder. Fr1 clonal band at range between 310-380 were excised with a clean scalpel followed by gel purification using QIAquick Gel Extraction kit (Qiagen, Valencia, CA). The concentration of DNA were measured by Qubit (Life technologies, Grand Island, NY), and the size of PCR amplicon was determined by 2100 Bioanalyzer (Agilent, Santa Clara, CA).

DLBCL samples which could not be amplified by IgVHFR1, IgVHFR2 assay was performed as an alternative using Tube B of IGH Gene Clonality assay (Cat. No. 1-10-0020) from InvivoScribe Technologies (San Diego, CA) according to the manufacturer's instruction. This master mix contains7 VH -FR2 primers + JH consensus primers, which target the framework 2 region within the variable region, and the joining region of the Ig heavy chain locus. FR2 fragment PCR reaction was set up and amplified using thermocycler program as described for FR1 fragment amplification. The quality controls for this assay were polyclonal control (IVS-0000), clonal

control (IVS-0030), no template control and DNA specimen ladder. Fr2 clonal band at range between 250-295 were excised with a clean scalpel followed by gel purification using QIAquick Gel Extraction kit (Qiagen, Valencia, CA). The concentration of DNA were measured by Qubit (Life technologies, Grand Island, NY), and the size of PCR amplicon was determined by 2100 Bioanalyzer (Agilent, Santa Clara, CA).

DNA library construction and targeted PCR Amplicon sequencing

DNA library was generated with 10 ng DNA input using illumina Truseq DNA Sample Preparation kit v2 (illumina, San Diego, CA) per manufacturer's instruction. The DNA library was quantified using Agilent Technologies 2100 Bioanalyzer (Agilent, Santa Clara, CA) for product size, or using Qubit for product concentration. For each Miseq run, 10 libraries were pooled together at 10 nM, and each sample was tagged with a unique index.

$V_HD_HJ_H$ Mapping and Data Analysis

The MiSeq runs produced 150bp paired-end sequence reads which were mapped to the human *IGH* BCR heavy chain reference database available from the IMGT website (ref. 1) using a modified nucleotide blast search similar to the previous published method (ref. 2). Read pairs that did not map to both an *IGH* V gene and *IGH* J gene were then discarded from further analysis. For each paired tumor and BM sample, the count of the number of paired end reads mapping to a particular V_H-J_H combination was taken and tabulated.

After the table of V_H-J_H genes were ranked by count in the tumor sample the V_H-J_H region with the highest count of read-pairs in the tumor sample was defined as the major rearrangement combination (accounting for mean 66.7% of all mapped V_H-J_H read-pairs over tumor samples and 20.96% in the paired BM samples), the subset of read pairs mapping to the major rearrangement combination in each tumor and accompanying paired BM/PBL sample were then

extracted and aligned to the major V_H-J_H sequence, discarding any sequences covering less than 80% of the reference sequence, producing a set of subclones denoted by their somatic hypermutation pattern against the reference, with their subclone counts for both the paired tumor and BM/PBL samples.

The patterns of SHM in the subclones were then used to draw phylogenetic trees of the matched tumor and BM/PBL samples in the newick tree format using the R package 'ape' (ref. 3) and then drawn as a rooted trees with subclone counts and SHM patterns shown using a custom perl script. To ensure the results were viewable the graphical display of the trees chose the 10 most abundant clones in both the tumor and BM samples and any subclones that overlapped between samples along with a random selection of minor subclones from all samples. Any further statistical testing and analysis was performed in R.

2. Supplementary Figure Legends

Supp. Figure 1.

Phylogenetic trees comparing tumor-related or major clones detected in the bone marrow (BM) and the diagnostic diffuse large B-cell lymphomas (DLBCLs) in patients without morphologic evidence of lymphomatous involvement in BM. Divergent/ancestral (DA) clones are marked by solid bars; Identical (I) clones are marked by dashed bars. The dominant tumor clone in the nodal/extranodal DLBCL is indicated by an asterisk.

Supp. Figure 2.

Phylogenetic trees comparing tumor-related clones detected in the bone marrow (BM) and the diagnostic diffuse large B-cell lymphomas (DLBCLs) in patients with morphologic evidence of

lymphomatous involvement in BM. Divergent/ancestral (DA) clones are marked by solid bars; Identical (I) clones are marked by dashed bars. The dominant tumor clone in the nodal/extranodal DLBCL is indicated by an asterisk.

Supp. Figure 3

Identification of chemo-resistant tumor clones ancestral or highly related to the relapse clones in post-treatment BM.

(A) Timeline of the diagnoses samples and treatment was illustrated in patient #22.

(B) In patient #22, minor ancestral/divergent tumor clones similar to those found in BM-1 and BM-2 of patient #45 were identified in both the post-transplant negative BM as well as in the original diagnostic DLBCL, implying that these minor tumor clones in the BM are chemo-resistant. Since the material for the relapsed DLBCL is not available for analysis, the relationship between these minor DA-type tumor clones in the marrow and the relapsed DLBCL cannot be determined.

Supp. Figure 4

A hypothetical model of bone marrow involvement in patients with DLBCL.

An ancestral clone in the lymph node (LN) gives rise to both the major dominant tumor clone(s) as well as the minor divergent clone(s). Dissemination to the BM of tumor cells derived from the ancestral/divergent or dominant clones in the LN results in the presence of DA-type and/or I-type clones in the BM, respectively. Occasionally, it is not possible to detect similar counterparts of the BM DA-type clones in the LN (dashed arrows). Initially, these clones in the BM are minute in quantity and only detectable by deep VDJ sequencing. However, these clones may expand and eventually be recognized as lymphomas based on morphological basis. In

several BMs we could see a dominant DA-type tumor clone co-existing with either minor or co-dominant I-type tumor clones. Presumably, these BMs are initially involved by minute tumor clones of both DA- and I-type, with subsequent different trajectories of clonal expansion in these tumors.

References:

1. The international Immunogenetics Information System www. Imgt.org

2. Deep sequencing reveals clonal evolution patterns and mutation events associated with relapse in B-cell lymphoma. Jiang, Redmond et al., Genome Biology 2014, 15:432

3. Ape: Analysis of Phylogenetics and Evolution. http://cran.r-project.org/web/packages/ape/index.thml
